# Novel Textbook Outcomes following emergency laparotomy: Delphi exercise

**DOI:** 10.1093/bjsopen/zrad145

**Published:** 2024-01-29

**Authors:** David N Naumann, Aneel Bhangu, Adam Brooks, Matthew Martin, Bryan A Cotton, Mansoor Khan, Mark J Midwinter, Lyndsay Pearce, Douglas M Bowley, John B Holcomb, Ewen A Griffiths, BEACON Collaborative, Adam Abu-Abeid, Adam Brooks, Adam Peckham-Cooper, Adam R Dyas, Ademola Adeyeye, Agron Dogjani, Alasdair C Y Ball, Albert M Wolthuis, Alejandro Quiroga-Garza, Aleksandar R Karamarkovic, Alessio Giordano, Alexander Fuchs, Alexander Julianov, Alexander W Phillips, Alexander Zimmermann, Alexandros Charalabopoulos, Alexei A Birkun, Alexis Rafael Narvaez-Rojas, Ali Guner, Amelia L Davis, Andras Vereczkei, Andrea Balla, Andrea Celotti, Andrea Romanzi, Andrea Trombetta, Andrew D Beggs, Andrew G Robertson, Andrew Petrosoniak, Andrew R Davies, Ángel Becerra-Bolaños, Anthony Loria, Antonio Brillantino, Antonios Athanasiou, Arda Isik, Argyrios Ioannidis, Ariel P Santos, Arin K Saha, Arturo Vilches-Moraga, Asad J Choudhry, Asuka Tsuchiya, B Mark Smithers, Bas P L Wijnhoven, B D Keeler, Belinda De Simone, Rodica Birla, Biswadev Mitra, Boyko Chavdarov Atanasov, Brian Badgwell, Brodie Nolan, Bryan A Cotton, Byung Hee Kang, Caoimhe C Duffy, Carlos A Ordoñez, Carlos Augusto Gomes, Carmen L Mueller, Caroline E Reinke, Carter C Lebares, Catherine J Hunter, Celia Villodre, Cem E Guldogan, Charalampos Seretis, Charles A Adams, Charles H C Pilgrim, Chris Varghese, Christian Owoo, Christian S Meyhoff, Christina A Fleming, Christina M Stuart, Christopher A Lewis-Lloyd, Christopher J McLaughlin, Claire L Stevens, Colin A Graham, Conor Magee, David I Saunders, D Dante Yeh, Daniel L Chan, Daniel M Felsenreich, Daniel N Holena, Dauda Bawa, David J Bowrey, David N Naumann, David S Liu, David S Y Chan, Deb Sanjay Nag, Diane N Haddad, Diletta Corallino, Dimitrios Damaskos, Dimitrios Moris, Dimitrios Schizas, Dimitris P Korkolis, Dinesh Kumar Bagaria, Dmitry Mikhailovich Adamovich, Douglas A Colquhoun, Douglas M Bowley, Dinesh Singhal, Manjunath Siddaiah-Subramanya, Rohit Kapoor, Duncan Wyncoll, Duong Van Hai, Ewoud Ter Avest, Edoardo Maria Muttillo, Edoardo Picetti, Edward Kelly, Efstratia Baili, Eleonora Pinto, Elif Colak, Elijah Dixon, Elisa Reitano, Emiko Sultana, Emily C Mills, Eric J Ley, Erik Osterman, Evan G Pivalizza, Evripidis Tokidis, Ewen A Griffiths, Anne-Cécile Ezanno, Fausto Catena, Federica Pederiva, Federico Coccolini, Felix Nickel, Ferdinando Agresta, Fernando Navarro Tovar, Fikri M Abu-Zidan, Filip Brzeszczyński, Michael El Boghdady, Flavio Roberto Takeda, Francesco Fleres, Francesca Pecchini, Francesco Maria Carrano, Francesco Pata, Francesk Mulita, Fredrik Klevebro, Gabriel Rodrigues, Gaetano Gallo, Gaetano Poillucci, Gary Alan Bass, Geeta Aggarwal, Gennaro Perrone, Geoffrey Roberts, Georgios Koukoulis, Georgios Zacharis, Gian Luca Baiocchi, Gianluca Pellino, Giorgio Lisi, Giovanni Dapri, Giuseppe Brisinda, Goran Augustin, Grigorios Christodoulidis, Guglielmo Imbriaco, Guillaume Ducarme, H Kemal Rasa, Peter W Hamer, Hans Lederhuber, Haralds Plaudis, Hayaki Uchino, Hazem Beji, Henry J M Ferguson, Hugo M L Cohen, Iain Wilson, Igor A Kryvoruchko, Ilari Kuitunen, Ilaria Benzoni, Ilenia Merlini, Ilze Ose, Imtiaz Wani, Ines Gockel, Ionut Negoi, Irena Gribovskaja-Rupp, Ivan Tomasi, Iyiade Olatunde Olaoye, J Cleo Kenington, J Scott Roth, Jacob Rosenberg, Jacopo Viganò, James Matthew Lloyd Williamson, Jan J De Waele, Jason E Smith, Jeffry Nahmias, Jennifer L Stevens, Jennifer Rickard, Jin Jiun Mah, Job F Waalwijk, Jonathan B Yuval, Joonas H Kauppila, Joseph Cuschieri, Joshua B Brown, Juan Gomez Rivas, Juliet Emamaullee, K Lasithiotakis, Katherine McKenzie, Kazuhide Matsushima, A I Koivusalo, L Max Almond, Lars Konge, Lars N Jorgensen, Laurent Genser, Lena M Napolitano, Leo R Brown, Lewis J Kaplan, Luca Degrate, Luigi Bonavina, Lynne Moore, Mahir Gachabayov, Mamun David Dornseifer, Manjunath Siddaiah-Subramanya, Mansour Abdulshafea, Marcelo A F Ribeiro Junior, Marcello Migliore, Marco Ceresoli, Marco Clementi, Marco Scarpa, Maria Olausson, Mariana R F Sousa, Mario Giuffrida, Mario D'Oria, Mario Pacilli, Martin Czerny, Martin Reichert, Martin Rutegård, Maryam Bahreini, Matthew J Lee, Matthew J Martin, Matti Tolonen, Matyas Fehervari, Maurizio Rho, Mauro Podda, Maxime Léger, Maximos Frountzas, Meer M Chisthi, Meghan R Lewis, Mélanie Bérubé, Melissa Oliveira-Cunha, Max E R Marsden, Mesut Tez, Micaela Piccoli, Michael F Bath, Michael Flanagan, Michael Gottlieb, Michael L Pearl, Michael P Achiam, Michael Swart, Mika Ukkonen, Miklosh Bala, Mohamed Ebrahim, Mohammed N AlAli, Monica Ortenzi, Montassar Ghalleb, Morten Hylander Møller, Muhammad R Iqbal, Muhammed A Ali, Munir Tarazi, Nicholas J Newton, Nader M Hanna, Nadia A Henriksen, Natalie S Blencowe, Neil Merrett, Neil T Welch, Nicola Colucci, Nicola de'Angelis, Nicola Latronico, Nicole L Werner, Niels D Martin, Nikolaos Machairas, Nikolay Bugaev, Ning Qi Pang, Obinna Obinwa, Onigbinde Oluwanisola Akanji, Panagiotis Kapsampelis, Paola De Nardi, Paolo Vincenzi, Patricio Lamoza Kohan, Philip H Pucher, Philip J J Herrod, Philip W Y CHIU, Pierluigi Marzuillo, Pierpaolo Sileri, Pietro Fransvea, Pradeep H Navsaria, Predescu Dragos Valentin, Roel Bakx, Rachel L Choron, Rahul Gupta, Rao R Ivatury, Raquel Diaz, Rebecca Anne Bradley, Reitano Elisa, René M Palacios Huatuco, Reza Shahriarirad, Rishi Rattan, Riyad Karmy-Jones, Robert G Sawyer, Robert J S Coelen, Roberto Cirocchi, Rondi B Gelbard, Roxanna Zakeri, Rui Farinha, Rutger M Schols, Ryan P Dumas, Salomone Di Saverio, Samik Kumar Bandyopadhyay, Samir Delibegovic, Sean Stevens, Sergio M Navarro, Shamita Chatterjee, Stamatios Petousis, Stavros Gourgiotis, Stephanie M Streit, Suman Baral, Sunaina T Karna, Susan Moug, Susan Yoong, Suzanne S Gisbertz, Tareq Kheirbek, Teoh Yuen-Chun Jeremy, Therese M Duane, Thomas Korgaard Jensen, Tim Bright, Timothy Craig Hardcastle, Triantafyllou Tania, Vahagn C Nikolian, Valentina Bianchi, Victor Kong, Vincenzo Trapani, Vishal G Shelat, Vishnu R Mani, Vladimir M Khokha, Wah Yang, Waleed Al-Khyatt, Yick Ho Lam, Yu Kijima, Yunfeng Cui, Zane B Perkins, Zaza Demetrashvili, Zi Qin Ng

**Affiliations:** Department of Trauma and Emergency General Surgery, University Hospitals Birmingham NHS Foundation Trust, Birmingham, UK; Department of Trauma and Emergency General Surgery, University Hospitals Birmingham NHS Foundation Trust, Birmingham, UK; NIHR Global Health Unit on Global Surgery, Institute of Translational Medicine, University of Birmingham, Birmingham, UK; East Midlands Major Trauma Centre, Queen's Medical Centre, Nottingham, UK; Division of Trauma and Acute Care Surgery, Department of Surgery, Los Angeles County & USC Medical Center, Los Angeles, California, USA; The Center for Translational Injury Research, McGovern Medical School at The University of Texas Health Science Center at Houston, Houston, Texas, USA; Department of General Surgery, University Hospitals Sussex NHS Foundation Trust, Brighton, UK; School of Biomedical Sciences, The University of Queensland, Brisbane, Queensland, Australia; Department of General Surgery, Salford Royal NHS Foundation Trust, Salford, UK; Department of Trauma and Emergency General Surgery, University Hospitals Birmingham NHS Foundation Trust, Birmingham, UK; Division of Acute Care Surgery, Department of Surgery, University of Alabama at Birmingham, Birmingham, Alabama, USA; Department of Trauma and Emergency General Surgery, University Hospitals Birmingham NHS Foundation Trust, Birmingham, UK

## Abstract

**Background:**

Textbook outcomes are composite outcome measures that reflect the ideal overall experience for patients. There are many of these in the elective surgery literature but no textbook outcomes have been proposed for patients following emergency laparotomy. The aim was to achieve international consensus amongst experts and patients for the best Textbook Outcomes for non-trauma and trauma emergency laparotomy.

**Methods:**

A modified Delphi exercise was undertaken with three planned rounds to achieve consensus regarding the best Textbook Outcomes based on the category, number and importance (Likert scale of 1–5) of individual outcome measures. There were separate questions for non-trauma and trauma. A patient engagement exercise was undertaken after round 2 to inform the final round.

**Results:**

A total of 337 participants from 53 countries participated in all three rounds of the exercise. The final Textbook Outcomes were divided into ‘early’ and ‘longer-term’. For non-trauma patients the proposed early Textbook Outcome was ‘Discharged from hospital without serious postoperative complications (Clavien–Dindo ≥ grade III; including intra-abdominal sepsis, organ failure, unplanned re-operation or death). For trauma patients it was ‘Discharged from hospital without unexpected transfusion after haemostasis, and no serious postoperative complications (adapted Clavien–Dindo for trauma ≥ grade III; including intra-abdominal sepsis, organ failure, unplanned re-operation on or death)’. The longer-term Textbook Outcome for both non-trauma and trauma was ‘Achieved the early Textbook Outcome, and restoration of baseline quality of life at 1 year’.

**Conclusion:**

Early and longer-term Textbook Outcomes have been agreed by an international consensus of experts for non-trauma and trauma emergency laparotomy. These now require clinical validation with patient data.

## Teaser

We propose novel Textbook Outcomes for emergency laparotomy (for trauma and non-trauma) based on a large international Delphi exercise

## Introduction

There has been a recent increase in interest in the use of Textbook Outcomes (TOs) for elective surgery, including colorectal^1^, oesophagogastric^2^, bariatric^3^, hepatobiliary^4^, gynaecological^5^ and cardiac^6^ surgery. These TOs are composite outcome measures that seek to better describe the ‘ideal’ outcome for patients. Rather than using single outcome measures such as ‘survival’ or ‘length of stay’, an example of a TO may be ‘survival following radical resection with no major complications, no reintervention, no unplanned stoma and no prolonged stay or readmission’^7^. TOs may therefore be more patient-centred, as well as enabling centres to assess performance^2^, develop new programmes^8^ and investigate risk factors for poorer outcomes^9^.

The majority of TOs have been designed for elective surgery, and there has been little attention towards TOs in the trauma and emergency surgery setting. The global burdens of morbidity rate and mortality rate from emergency surgical conditions are highly significant and have been neglected for many years, with millions of disability-adjusted-life years lost from 11 Emergency General Surgery conditions alone^10^. The Global Burden of Disease (GBD) study showed that in 2019, 8% of all deaths worldwide were due to injury^11^, and while it is not known what proportion of severely injured patients require emergency laparotomy, when this has been scrutinized at a national level, trauma emergency laparotomy has been found to be both highly morbid and a major drain on resources^12^. Focus on outcomes after trauma emergency laparotomy is particularly important, since these can be obscured when larger cohorts of ‘injured patients’ are evaluated^13^.

It is timely for attention to be paid to the design and investigation of TOs following trauma and non-trauma emergency laparotomy, and this should be a focus for the improvement of patient care and for capturing relevant global outcomes that have a high impact on the postoperative functional status and quality of life (QoL)^14^.

The aim of the current Delphi exercise was to achieve consensus from an international panel of experts on what might be considered the optimal TO following trauma and non-trauma emergency laparotomy.

## Methods

### Study design

An international Delphi exercise was undertaken to survey a large and diverse group of surgical experts in both trauma and non-trauma emergency laparotomy. This was undertaken using online survey software distributed in three planned rounds. An interactive session with a patient and public focus group was also used to inform the Delphi exercise and provide patient-centred input.

### Participant selection

Participants were selected for the Delphi exercise based on their expertise in the care for patients undergoing trauma and non-trauma emergency laparotomy. A search was made using PubMed for published articles relating to laparotomy using search terms such as ‘laparotomy’, ‘emergency laparotomy’ and ‘trauma laparotomy’. The corresponding authors for relevant articles were e-mailed a generic invitation to participate in the first round of the Delphi exercise. A link to the survey was also distributed on social media for users who provide surgical care for this patient population. Although there was an expectation that most respondents would be practicing surgeons, the responses of non-surgeons were also welcomed. There was, therefore, a domain in the survey for ‘role in healthcare’ so that this information could be collected.

### Delphi exercise

There were three planned rounds of the Delphi exercise, starting with very broad questions, and then narrowing down the questions in subsequent iterations. Participants were asked whether they agreed with statements using a Likert scale (1–5, corresponding to ‘Strongly disagree’, ‘Disagree’, ‘Neutral’, ‘Agree’ and ‘Strongly agree’). Questions related to which individual outcomes should be included in the TO, how long the follow-up interval should be and how many outcome measures could be included in the TO. The results of the previous rounds were displayed in each iteration (the full surveys are shown in *Results S1–3*) so that participants were able to adjust their answers based on previous results from the whole group. Each survey reiterated the aims and rationale for the exercise and asked the participants to acknowledge their understanding. Participants were masked to each other’s identity and responses, and analysis was undertaken masked to the participants. However, names and e-mail address details were recorded centrally to distribute subsequent rounds of the exercise and to inform the collective authorship. Invitations to participate in rounds 2 and 3 of the Delphi exercise were sent to the e-mail addresses of participants who had indicated in previous rounds that they wished to continue to participate. Two subsequent targeted follow-up e-mails were sent for non-responders before moving on to the next round.

### Patient and public exercise

After round 2 of the Delphi exercise, a public and patient involvement (PPI) focus group exercise was undertaken to better inform round 3 of the exercise. This was an experienced PPI group that has participated in multiple studies and is based at the Trauma, Accident, Burns and Critical Care Group (TrABC), sponsored by the National Institute of Health Research, UK. This was planned for this stage of the Delphi exercise so that the group could see what answers had already been given, for points of focused discussion. These were discussed in sequence and in detail, and the opinions of the group were sought on these topics.

### Data analysis

Data were summarized using median and interquartile range for continuous data and number and percentage for categorical data. A map of worldwide participation was created using Maptive (Maptive^TM^, San Francisco, CA, USA).

## Results

### Delphi exercise participants

**Fig. 1:**
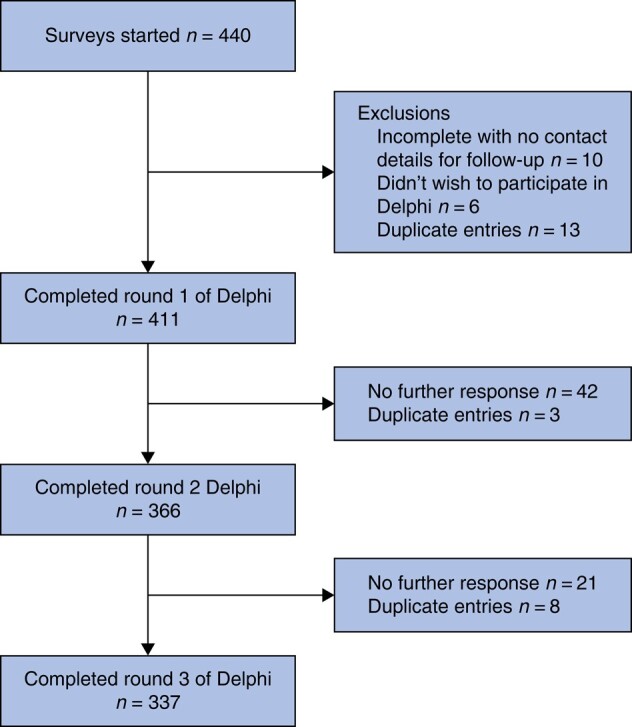
Flow diagram of participation in the Delphi exercise

**Fig. 2:**
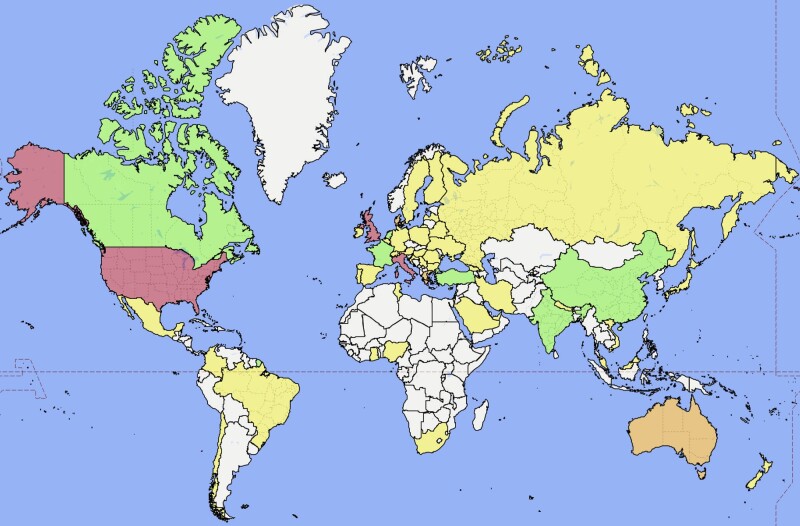
Representation of countries worldwide (yellow indicates 1–5; green 6–10; orange 11–30; red 31+ participants)

There were 411 participants who completed round 1 of the Delphi exercise, 366 who completed round 2 and 337 participants who completed all 3 rounds. *Figure 1* illustrates the participation at each round. Characteristics of participants are summarized in *Table 1*. Most participants were Attending/Consultant grade surgeons, and 79.5% were male. This was an experienced cohort, with most participants regularly performing both non-trauma and trauma emergency laparotomy, although the former was more commonly performed. There was representation from 53 countries worldwide (*Fig. 2*). The full list of participating countries is in *Table S1*.

**Table 1:** Characteristics of Delphi participants

Characteristic	Summary (*n* = 337)
**Role in healthcare**	
Medically trained, Attending/Consultant	266 (78.9)
Medically trained, non-Attending/Consultant	54 (16.0)
Researcher	10 (3.0)
Other	7 (2.1)
**Sex**	
Male	268 (79.5)
Female	63 (18.7)
Other/not declared	6 (1.8)
Year of graduation from medical school, median (i.q.r.)	2005 (1998–2011)
Regularly perform non-trauma laparotomy	298 (88.4)
Regularly perform trauma laparotomy	227 (67.4)
**Total number of non-trauma laparotomies performed**	
<100	133 (39.5)
100–300	105 (31.2)
>300	96 (28.5)
Not declared	4 (1.2)
**Total number of trauma laparotomies performed**	
<100	228 (67.7)
100–300	61 (18.1)
>300	41 (12.2)
Not declared	7 (2.1)

Values are *n* (%) unless otherwise stated.

### Individual outcome measures

When participants were asked about individual outcome measures that should be included in the TOs for trauma and non-trauma emergency laparotomy, the answers with most scores of 5 (‘strongly agree’) were ‘postop complications’, ‘death/survival’, ‘intra-abdominal sepsis/fistula/leak/abscess’, ‘unplanned re-operation on’, ‘organ failure’ and ‘QoL’ for both types of surgery. In addition, for emergency trauma laparotomy, ‘blood products used’ also had most scores of 5. These were also the outcome measures most favoured by the PPI group. When asked how many outcome measures should be incorporated into the TOs, the most common answer was ‘5’. When the PPI group was asked about which QoL measure should be used, they were generally not in favour of long or arduous surveys and would prefer that the QoL measure was more general, such as a patient-reported outcome measure (PROM) ‘subjective return to baseline QoL’. This was agreed by the majority of Delphi participants in round 3 of the exercise (284 of 337 (84.3%)).

### Follow-up requirement

When participants were asked about follow-up interval, the category with the highest number of scores of 5 was ‘1 year’. When the PPI group was consulted, they stated that it would be a good idea to split the TO into ‘short-term’ and ‘long-term’ TOs so that follow-up could be 1 year, but that the shorter-term outcomes should also be recognized. In round 3 of the Delphi exercise this was widely agreed by participants as a good idea (305 of 337 (90.5%)).

### Textbook Outcomes

The final agreed TOs for trauma and non-trauma emergency laparotomy after round 3 of the Delphi were divided into Early Textbook Outcome (E-TO) and Longer-Term Textbook Outcome (LT-TO) and are summarized in *Table 2* according to the Delphi exercise. Individual complications were defined according to well-established Clavien–Dindo definitions^15^ that are familiar to surgeons and were therefore not re-defined in this exercise.

**Table 2:** Textbook Outcomes for non-trauma and trauma laparotomy after round 3 of the Delphi exercise

Category of surgery	Textbook Outcome definition	Agreed without further comment
**Non-trauma laparotomy**		
* *Early	Discharged from hospital without serious postoperative complications (that is Clavien–Dindo ≥ grade III; including intra-abdominal sepsis, organ failure, unplanned re-operation on or death)	294 (87.2)
* *Longer-term	Achieved the E-TO, and restoration of baseline quality of life at 1 year	
**Trauma laparotomy**		
* *Early	Discharged from hospital without unexpected transfusion after haemostasis, and no serious postoperative complications (adapted Clavien–Dindo for trauma ≥ grade III; including intra-abdominal sepsis, organ failure, unplanned re-operation on or death)	284 (84.3)
* *Longer-term	Achieved the E-TO, and restoration of baseline quality of life at 1 year	

Values are *n* (%). E-TO, early textbook outcome.

### Disagreements

As might be expected in a group of diverse contributors including surgeons, there was imperfect consensus amongst the 337 participants. There were six participants that stated that these TOs were too complicated to measure. There were four participants who stated that they thought that 1 year was too long for the LT-TO due to the risk of patients lost to follow-up. There were eight participants who did not think that restoration of baseline QoL was a good measure for the LT-TO. Some of these participants stated that there should be a more specific and objective QoL assessment, and others stated that this was an unrealistic expectation that may result in very few patients achieving the LT-TO. There were three participants who felt that incisional hernia should feature in the TOs.

## Discussion

After a Delphi exercise and engagement with a PPI group, consensus has been achieved on both early and longer-term TOs following trauma and non-trauma emergency laparotomy for the purposes of patient-centred research for this important population. For non-trauma emergency laparotomy, the E-TO proposed is: ‘Discharged from hospital without serious postoperative complications (that is Clavien–Dindo^15^ ≥ grade III; including intra-abdominal sepsis, organ failure, unplanned re-operation on or death)’. For trauma laparotomy: ‘Discharged from hospital without unexpected transfusion after haemostasis, and no serious postoperative complications (adapted Clavien–Dindo for trauma^16^ ≥ grade III; including intra-abdominal sepsis, organ failure, unplanned re-operation on or death).’ For both trauma and non-trauma emergency laparotomy, an LT-TO of ‘Achieved the E-TO, and also patient-reported restoration to their baseline QoL at 1 year’ is proposed.

These represent the first proposals for TOs for patients following trauma and non-trauma emergency laparotomy. Designing TOs for other than elective surgery is complicated due to a higher variability in presentations, co-morbidities, pathologies, interventions, and participation in both short- and long-term follow-up. However, this does not mean that TOs should not be designed or implemented, and in fact argues for the critical necessity of establishing TOs in this patient population. Research in trauma and emergency surgery has historically been relatively neglected even though it has an enormous impact on the global burden of disease^10^. To determine whether these TOs will be of use in future research for patients following trauma and non-trauma emergency laparotomy, it will be important to validate the TOs using patient data^14^. Ideally this should be done on an international scale, as well as for individual institutions and systems. Such an exercise may provide evidence that the data is feasible to obtain, and that there is correlation with other outcomes of interest. If validated, these TOs should be considered for usage as primary outcomes for prospective clinical trials and potentially incorporated into national trauma and emergency general surgery registries focused on quality. Examples may include the Trauma Quality Improvement Program in the USA and the National Emergency Laparotomy Audit in the UK.

It is notable that the LT-TO for both trauma and non-trauma emergency laparotomy includes a subjective measure of restoration of QoL to baseline as a patient-reported measure, without a specific tool or instrument mandated. This represents an opportunity for researchers to have the freedom to choose whichever PROM they wish, as long as it is able to reliably determine and quantify the restoration of baseline QoL. This is an important aspect of this TO since there are already many PROMs that have been proposed^17,18^. A recent trauma trial for patients with haemorrhagic shock used the primary outcome of survival ‘with favourable functional outcome at 6 months’^19^. This is a good modern example of such a PROM being used to form a composite outcome and demonstrates the current desire for new trials to use more patient-centred outcomes. It is not the role of a TO to determine the precise tools of measurement, and it is recommended that future studies consult PPI groups to agree which PROM would best-suit the research question at hand and the specific population being studied. There may also be major differences in the ideal QoL metrics or sampling methodology between patient populations based on demographic factors such as nationality, race, ethnicity, gender, income, education level and similar variables. Having the flexibility to adopt the specific PROM methodology to the population being studied may result in greater patient response, diversity of responses and inclusivity.

The proposed LT-TO requires follow-up at 1 year, which may have its own issues regarding patients being lost to follow-up, due to change in contact details or address, loss of details or disengagement. A considerable proportion of patients following trauma and non-trauma emergency laparotomy may have also died within such a follow-up interval^20,21^. Patients lost to follow-up may be demographically different to those with follow-up data^22,23^. However, improvement of follow-up can be achieved by improving systems^24^, and identification of risk factors for loss to follow-up may be useful to implement a targeted approach^25^. The requirement for a 1-year review for research may enable centres and systems to improve their longer-term follow-up data collection and processes, which would be a useful change for patient care. The patient and public focus group were strongly of the opinion that there should be a longer-term review of patients after trauma and non-trauma emergency laparotomy, and this is likely to be reflected in the opinions of other patients. It is recommended that researchers choosing the LT-OT as an outcome measure in the methodology of prospective studies ensure they design robust strategies to better enable 12-month follow-up.

It is acknowledged that for many patients who have an emergency laparotomy, obtaining a TO may never be achievable due to the nature of the physiological insults prior to surgery. This is different to elective surgery, where surgeons might more realistically aim for 100% of their patients to achieve a TO. However, this limitation is shared by most outcome measures used for emergency surgery, where a certain level of poorer outcomes are expected regardless of the quality of care. This makes the design of TOs for emergency surgery different to elective surgery. Nevertheless, such outcome measures are justified because they can be used for purposes such as monitoring quality of care over time, between centres or following novel treatments.

Although more than 84% consensus was achieved for the final TOs, this Delphi process also identified areas of imperfect consensus surrounding perceived complexity of the outcome measures; appropriate length of follow-up; agreement on the definition of what constitutes restoration of QoL, and consideration for inclusion of incisional hernia as a specific (poor) outcome after trauma and non-trauma emergency laparotomy. Achieving perfect consensus on these complex issues is considered unrealistic, and less desirable than having a broad range of opinions and input in the development of robust TOs. Further refinement of these TOs may be desirable in the future. Other limitations include acknowledgement of a relative under-representation of lower-income countries, such as within the African continent. There was also an under-representation of female participants (only 18.7% in this study), which may reflect current overall gender disparities amongst surgeons worldwide. Participants were not asked for their surgical specialty, but instead used the data about numbers of laparotomies that they had performed as a marker of their expertise in the subject. It was not, therefore, possible to summarize the specialties for better characterization of participating surgeons. Since social media was used to amplify the distribution of the invitation to participate, the true denominator of those who had seen the invitation is unknown. It is therefore not possible to report the true proportion of potential participants (invitees) who participated in the Delphi exercise.

There is agreement amongst a large international group of surgeons, other healthcare professionals and patients about which TOs can be proposed for clinical research that includes patients following trauma and non-trauma emergency laparotomy. These are divided into short-term and longer-term TOs and can be used as primary outcome measures to investigate treatments and optimize care for these patients. The proposed TOs now need to be validated to be considered useful for this purpose and promulgated locally and worldwide.

## Collaborators

Adam Abu-Abeid (Tel-Aviv Sourasky Medical Center, Tel-Aviv, Israel); Adam Brooks (East Midlands Major Trauma Centre, Nottingham, UK); Adam Peckham-Cooper (Leeds Institute of Emergency General Surgery, Leeds, UK); Adam R. Dyas (University of Colorado, Denver, CO, USA); Ademola Adeyeye (Afe Babalola University, Ado-Ekiti, Nigeria); Agron Dogjani (University of Medicine of Tirana, Tirana, Albania); Alasdair C.Y. Ball (Birmingham Heartlands Hospital, Birmingham, UK); Albert M. Wolthuis (University Hospital Leuven, Leuven, Belgium); Alejandro Quiroga-Garza (Universidad Autónoma de Nuevo León, Monterrey, Mexico); Aleksandar R. Karamarkovic (University of Belgrade, Belgrade, Serbia); Alessio Giordano (Careggi University Hospital, Florence, Italy); Alexander Fuchs (University of Bern, Bern, Switzerland); Alexander Julianov (Trakia Hospital, Stara Zagora, Bulgaria); Alexander W. Phillips (Northern Oesophagogastric Unit, Newcastle upon Tyne, UK); Alexander Zimmermann (University Hospital Zurich, Zurich, Switzerland); Alexandros Charalabopoulos (Laiko General Hospital, Athens, Greece); Alexei A. Birkun (Crimean Federal University, Simferopol, Russian Federation); Alexis Rafael Narvaez-Rojas (University of Miami Miller School of Medicine, Miami, FL, USA); Ali Guner (Karadeniz Technical University, Trabzon, Turkey). Aly Fayed (Austin Health, Melbourne, Australia); Amelia L. Davis (Sir Charles Gairdner Hospital, Nedlands, Australia); Andras Vereczkei (University of Pécs, Pécs, Hungary); Andrea Balla (IRCCS San Raffaele Scientific Institute, Milan, Italy); Andrea Celotti (Ospedale Maggiore di Cremona, Cremona, Italy); Andrea Romanzi (Valduce Hospital, Como, Italy); Andrea Trombetta (University of Trieste, Trieste, Italy); Andrew D. Beggs (University Hospitals Birmingham, Birmingham, UK); Andrew G. Robertson (Royal Infirmary Edinburgh, Edinburgh, UK); Andrew Petrosoniak (St. Michael's Hospital, Toronto, Canada); Andrew R. Davies (Guy's & St Thomas' NHS Foundation Trust, London, UK); Ángel Becerra-Bolaños (Hospital Universitario de Gran Canaria Doctor Negrín, Spain); Anthony Loria (University of Rochester Medical Center, Rochester, NY, USA); Antonio Brillantino (A. Cardarelli hospital, Naples, Italy); Antonios Athanasiou (University Hospitals of Sussex, Brighton, UK); Arda Isik (Istanbul Medeniyet University, Istanbul, Turkey); Argyrios Ioannidis (Athens Medical Center, Athens, Greece); Ariel P. Santos (Texas Tech University Health Sciences Center, Lubbock, TX, USA); Arin K. Saha (Huddersfield Royal Infirmary, Huddersfield, UK); Arturo Vilches-Moraga (Hamad Medical Corporation, Doha, Qatar); Asad J. Choudhry (SUNY Upstate Medical University, Syracuse, NY, USA); Asuka Tsuchiya (Tokai University School of Medicine, Kanagawa, Japan); B. Mark Smithers (University of Queensland, Princess Alexandra Hospital, Brisbane, Australia); Bas P.L. Wijnhoven (Erasmus University Medical Center, Rotterdam, The Netherlands); B.D. Keeler (Milton Keynes University Hospital, Milton Keynes, UK); Belinda De Simone (Academic Hospital of Villeneuve St Georges, Paris, France); Rodica Birla (Carol Davila University Bucharest, Romania); Biswadev Mitra (The Alfred Hospital, Melbourne, Australia); Boyko Chavdarov Atanasov (Medical University of Plovdiv, Plovdiv, Bulgaria); Brian Badgwell (MD Anderson, Houston, TX, USA); Brodie Nolan (University of Toronto, Toronto, Canada); Bryan A. Cotton (McGovern Medical School at UTHealth, Houston, TX, USA); Byung Hee Kang (Ajou University School of Medicine, Suwon, Republic of Korea); Caoimhe C. Duffy (University of Pennsylvania, Philadelphia, PA, USA); Carlos A. Ordoñez (Universidad Icesi—Fundación Valle del Lili, Cali, Colombia); Carlos Augusto Gomes (Universidade Federal de Juiz de Fora, Juiz de Fora, Brazil); Carmen L. Mueller (Montreal General Hospital, Montreal, Canada); Caroline E. Reinke (Atrium Health, Charlotte, NC, USA); Carter C. Lebares (University of California San Francisco, San Francisco, CA, USA); Catherine J. Hunter (Oklahoma Children's Hospital, Oklahoma City, OK, USA); Celia Villodre (Hospital General Universitario Dr. Balmis, Alicante, Spain); Cem E. Guldogan (Qatar Turkish Hospital, Doha, Qatar); Charalampos Seretis (Agios Andreas General Hospital of Patras, Patras, Greece); Charles A. Adams Jr. (Warren Alpert Medical School of Brown University, Providence, RI, USA); Charles H.C. Pilgrim (The Alfred Hospital, Victoria, Australia); Chris Varghese (University of Auckland, Auckland, New Zealand); Christian Owoo (University of Ghana Medical School and Korle Bu Teaching Hospital, Accra, Ghana); Christian S. Meyhoff (Copenhagen University Hospital - Bispebjerg and Frederiksberg, Copenhagen, Denmark); Christina A. Fleming (University Hospital Limerick, Limerick, Ireland); Christina M. Stuart (University of Colorado, Aurora, CO, USA); Christopher A. Lewis-Lloyd (Nottingham University Hospitals NHS Trust, Nottingham, UK); Christopher J. McLaughlin (Penn State College of Medicine, Hershey, PA, USA); Claire L. Stevens (Southern Adelaide Local Health Network, SA Health, Australia); Colin A. Graham (Chinese University of Hong Kong, Prince of Wales Hospital, Shatin, Hong Kong SAR); Conor Magee (Wirral University Teaching Hospitals NHS Foundation Trust, Wirral, UK); David I. Saunders (Royal Victoria Infirmary, Newcastle upon Tyne, UK); D. Dante Yeh (University of Colorado, Denver, CO, USA); Daniel L. Chan (St George and Sutherland Clinical School, Faculty of Medicine and Health, University of New South Wales, Sydney, Australia); Daniel M. Felsenreich (Medical University of Vienna, Vienna, Austria); Daniel N. Holena (Medical College of Wisconsin, Milwaukee, WI, USA); Dauda Bawa (Haql General Hospital, Haql City, Saudi Arabia); David J. Bowrey (University Hospitals of Leicester NHS Trust, Leicester, UK); David N. Naumann (University Hospitals Birmingham, Birmingham, UK); David S. Liu (University of Melbourne, Department of Surgery, Austin Health, Heidelberg, Victoria, Australia); David S.Y. Chan (University Hospitals Plymouth NHS Trust, Plymouth, UK); Deb Sanjay Nag (Tata Main Hospital, Jamshedpur, India); Diane N. Haddad (University of Pennsylvania, Philadelphia, PA, USA); Diletta Corallino (Sapienza University of Rome, Rome, Italy); Dimitrios Damaskos (Royal Infirmary of Edinburgh, Edinburgh, UK); Dimitrios Moris (Duke University Medical Center, Durham, NC, USA); Dimitrios Schizas (National and Kapodistrian University of Athens, Athens, Greece); Dimitris P. Korkolis (Hellenic Anticancer Hospital, Greece); Dinesh Kumar Bagaria (All India Institute of Medical Sciences, New Delhi, India); Dmitry Mikhailovich Adamovich (Gomel State Medical University, Gomel, Belarus); Douglas A. Colquhoun (University of Michigan, Ann Arbor, MI, USA); Douglas M. Bowley (Royal Centre for Defence Medicine, Birmingham, UK); Dinesh Singhal (Max Super Speciality Hospital, New Delhi, India); Dr Manjunath Siddaiah-Subramanya (Bankstown Lidcombe Hospital, Sydney, Australia); Dr Rohit Kapoor (Barking Havering and Redbridge University Hospitals, UK); Duncan Wyncoll (Guy's & St Thomas' NHS Trust, London, UK); Duong Van Hai (University Medical Center, Hochiminh City, Vietnam); Ewoud Ter Avest (University Medical Center Groningen, University of Groningen, Groningen, The Netherlands); Edoardo Maria Muttillo (Sant'Andrea Hospital, Sapienza University of Rome, Rome, Italy); Edoardo Picetti (Parma University Hospital, Parma, Italy); Edward Kelly (UMass Chan School of Medicine- Baystate, Springfield, MA, USA); Efstratia Baili (Guy's and St Thomas' NHS Foundation Trust, London, UK); Eleonora Pinto (Veneto Institute of Oncology IOV - IRCCS, Padua, Italy); Elif Colak (University of Samsun, Turkey); Elijah Dixon (University of Calgary, Calgary, Canada); Elisa Reitano (Research Institute against Digestive Cancer, Strasbourg, France) Elisa Zambaiti (Ospedale Infantile Regina Margherita, Torino, Italy); Emiko Sultana (The Dudley Group NHS Foundation Trust, Dudley, UK); Emily C. Mills (Mid and South Essex Trust, Essex, UK); Eric J. Ley (Cedars-Sinai Medical Center, USA); Erik Osterman (Gävle Hospital, Gävle, Sweden); Evan G. Pivalizza (McGovern Medical School at UT Health Houston, Houston, TX, USA); Evripidis Tokidis (Sheffield Teaching Hospital NHS Trust, Sheffield, UK); Ewen A. Griffiths (University Hospitals Birmingham NHS Foundation Trust, Birmingham, UK); Anne-Cécile Ezanno (Begin Military Teaching Hospital, Saint Mandé, France); Fausto Catena (Bufalini Hospital, Cesena, Italy); Federica Pederiva (F. Del Ponte Hospital, Varese, Italy); Federico Coccolini (Pisa University Hospital, Pisa, Italy); Felix Nickel (University Medical Center Hamburg-Eppendorf, Hamburg, Germany); Ferdinando Agresta, (Presidio Ospedaliero di Vittorio Veneto, Italy); Fernando Navarro Tovar (Hospital Universitario de Puebla, Mexico); Fikri M. Abu-Zidan (United Arab Emirates University, Al-Ain, UAE); Filip Brzeszczyński (Medical University of Lodz, Poland); Michael El Boghdady (Guy's and St Thomas' NHS Foundation Trust, London, UK); Flavio Roberto Takeda (University of São Paulo, São Paulo, Brazil); Francesco Fleres (Department of Human Pathology of the Adult and Evolutive Age “Gaetano Barresi”, Section of General and Emergency Surgery, University of Messina, Messina, Italy); Francesca Pecchini (Baggiovara General Hospital, Modena, Italy); Francesco Maria Carrano (Busto Arsizio Circolo Hospital, Varese, Italy); Francesco Pata (University of Calabria, Rende, Italy); Francesk Mulita (General University Hospital of Patras, Patras, Greece); Fredrik Klevebro (Karolinska Institutet, Stockholm, Sweden); Gabriel Rodrigues (Aster Al Raffah Hospital, Sohar, Sultanate of Oman); Gaetano Gallo (Sapienza University of Rome, Rome, Italy); Gaetano Poillucci (Ospedale San Matteo degli Infermi, Spoleto, Italy); Gary Alan Bass (University of Pennsylvania, Philadelphia, PA, USA); Geeta Aggarwal (Royal Surrey Hospital NHS Foundation Trust, Guildford, UK); Gennaro Perrone (Parma University Hospital, Parma, Italy); Geoffrey Roberts (Oxford University Hospitals NHS Foundation Trust, Oxford, UK); Georgios Koukoulis (General Hospital of Larissa, Larissa, Greece); Georgios Zacharis (General Hospital St. Andrew Patras, Patra, Greece); Gian Luca Baiocchi (University of Brescia, Italy); Gianluca Pellino (Vall d'Hebron University Hospital, Universitat Autonoma de Barcelona UAB, Barcelona, Spain); Giorgio Lisi (Sant'Eugenio Hospital, Rome, Italy); Giovanni Dapri (Humanitas Gavazzeni University Hospital, Bergamo, Italy); Giuseppe Brisinda (Fondazione Policlinico Universitario A. Gemelli IRCCS, Rome, Italy); Goran Augustin (University Hospital Centre Zagreb, Zagreb, Croatia); Grigorios Christodoulidis (University Hospital of Larisa, Larisa, Greece); Guglielmo Imbriaco (Maggiore Hospital Carlo Alberto Pizzardi, Bologna, Italy); Guillaume Ducarme (Centre Hospitalier Departemental, La Roche sur Yon, France); H. Kemal Rasa (Anadolu Medical Center Hospital, Gebze Kocaeli, Turkey); Peter W. Hamer (University of Newcastle, Newcastle, NSW, Australia); Hans Lederhuber (Royal Devon University Healthcare NHS Foundation Trust, Exeter, UK); Haralds Plaudis (Riga East Clinical University Hospital, Riga, Latvia); Hayaki Uchino (McGill University, Montreal, Canada); Hazem Beji (Hospital Mohamed Taher Maamouri, Nabeul, Tunisia); Henry J.M. Ferguson (South Warwickshire University NHS Foundation Trust, UK); Hugo M.L. Cohen (HQ AMS Sp Unit, Camberley, UK); Iain Wilson (Frimley Health Foundation Trust, UK); Igor A. Kryvoruchko (Kharkiv National Medical University, Kharkiv, Ukraine); Ilari Kuitunen (University of Eastern Finland, Kuopio, Finland); Ilaria Benzoni (ASST Cremona, Italy); Ilenia Merlini (San Benedetto del Tronto Hospital, AST Ascoli Piceno, Italy); Ilze Ose (Zealand University Hospital, Denmark); Imtiaz Wani (Govt. Gousia Hospital, Srinagar, India); Ines Gockel (University Hospital Leipzig, Leipzig, Germany); Ionut Negoi (Carol Davila University of Medicine and Pharmacy Bucharest, Emergency Hospital of Bucharest, Bucharest, Romania); Irena Gribovskaja-Rupp (University of Iowa Hospitals & Clinics, Iowa City, IA, USA); Ivan Tomasi (Guys and St Thomas Hospital London, London, UK); Iyiade Olatunde Olaoye (University of Ilorin Teaching Hospital, Ilorin, Nigeria); J. Cleo Kenington (St George's Hospital, London, UK); J. Scott Roth (University of Kentucky, Lexington, KY, USA); Jacob Rosenberg (Herlev Hospital, University of Copenhagen, Denmark); Jacopo Viganò (Fondazione IRCCs Policlinico San Matteo, Pavia, Italy); James Matthew Lloyd Williamson (The Great Western Hospital, Swindon, UK); Jan J. De Waele (Ghent University Hospital, Ghent, Belgium); Jason E. Smith (Academic Department of Military Emergency Medicine, Birmingham, UK); Jeffry Nahmias (University of California Irvine, Orange, CA, USA); Jennifer L. Stevens (Ashford and St Peters Hospitals NHS Foundation Trust, UK); Jennifer Rickard (University of Minnesota, Minneapolis, MN, USA); Jin Jiun Mah (Queen Elizabeth Hospital, Kota Kinabalu, Malaysia); Job F. Waalwijk (University Medical Center Utrecht, Utrecht, The Netherlands). John V. Taylor (Aintree University Hospital, Liverpool, UK); Jonathan B. Yuval (Tel-Aviv Sourasky Medical Center, Tel-Aviv, Israel); Joonas H. Kauppila (Oulu University Hospital, Oulu, Finland); Joseph Cuschieri, (University of California San Francisco, San Francisco, CA, USA); Joshua B. Brown (University of Pittsburgh, Pittsburgh, PA, USA); Juan Gomez Rivas (Hospital Clinico San Carlos, Madrid, Spain); Juliet Emamaullee (University of Southern California, Los Angeles, CA, USA); K. Lasithiotakis (University Hospital of Heraklion, Greece); Katherine McKenzie (Jamaica Hospital Medical Center, New York, NY, USA); Kazuhide Matsushima (LAC+USC Medical Center, Los Angeles, CA, USA); Koivusalo A.I. (New Children's Hospital, Helsinki, Finland); L. Max Almond (University Hospital Birmingham, Birmingham, UK); Lars Konge (Copenhagen Academy for Medical Education and Simulation, Copenhagen, Denmark); Lars N. Jorgensen (Bispebjerg Hospital, Copenhagen, Denmark); Laurent Genser (Sorbonne Université, Assistance Publique- Hôpitaux de Paris, Paris, France); Lena M. Napolitano MD (University of Michigan, Ann Arbor, MI, USA); Leo R. Brown (Clinical Surgery, University of Edinburgh, Royal Infirmary of Edinburgh, Edinburgh, UK); Lewis J. Kaplan (University of Pennsylvania, Philadelphia, PA, USA); Luca Degrate (Fondazione IRCCS San Gerardo dei Tintori, Monza, Italy); Luigi Bonavina (University of Milan, Italy); Lynne Moore (Centre de Recherche du CHU de Québec, Québec City, Canada); Mahir Gachabayov (Westchester Medical Center, New York, NY, USA); Mamun David Dornseifer (North Middlesex University Hospital NHS Trust, London, UK); Manjunath Siddaiah-Subramanya (Bankstown Lidcombe Hospital, Sydney, Australia); Mansour Abdulshafea (Swansea Bay Health Board, Swansea, UK); Marcelo A.F. Ribeiro Junior (Sheikh Shakhbout Medical City, Abu Dhabi, UAE); Marcello Migliore (King Faisal Specialist Hospital & Research Center, Riyadh, Saudi Arabia); Marco Ceresoli (University of Milano-Bicocca, Monza, Italy); Marco Clementi (San Salvatore Hospital, University of L'Aquila, L'Aquila, Italy); Marco Scarpa (Azienda Ospedale Università di Padova, Padova, Italy); Maria Olausson (Zealand University Hospital, Koege, Denmark); Mariana R.F. Sousa (Hospital Beatriz Ângelo, Loures, Portugal); Mario Giuffrida (Parma University Hospital, Parma, Italy); Mario D'Oria (Department of Clinical Surgical and Health Sciences, University of Trieste, Trieste, Italy); Mario Pacilli (University of Foggia, Foggia, Italy); Martin Czerny (Albert Ludwigs University, Freiburg, Germany); Martin Reichert (Department for General, Visceral, Thoracic and Transplant Surgery, University Hospital of Giessen, Giessen, Germany); Martin Rutegård (Umeå University, Umeå, Sweden); Maryam Bahreini (Tehran University of Medical Sciences, Tehran, Iran) Matthew Forshaw (Glasgow Royal Infirmary, Glasgow, UK); Matthew J. Lee (University of Sheffield, Sheffield, UK); Matthew J. Martin (USC Medical Center, Los Angeles, CA, USA); Matti Tolonen (HUS Helsinki University Hospital, Helsinki, Finland); Matyas Fehervari (Imperial College London, London, UK); Maurizio Rho (Policlinico Tor Vergata, Rome, Italy); Mauro Podda (Cagliari State University, Italy); Maxime Léger (UCSF, San Francisco, CA, USA); Maximos Frountzas (National and Kapodistrian University of Athens, Athens, Greece); Meer M. Chisthi (Government Medical College, Konni, India); Meghan R. Lewis (Los Angeles General Medical Center+USC, Los Angeles, CA, USA); Mélanie Bérubé (Centre de recherche du CHU de Québec-Université Laval, Québec City, Canada); Melissa Oliveira-Cunha (University Hospitals of Birmingham, Birmingham UK); Max E.R. Marsden (Academic Department of Military Surgery and Trauma, Birmingham, UK); Mesut Tez (Ankara Numune Hospital, Turkey); Micaela Piccoli (Baggiovara General Hospital, Modena, Italy); Michael F. Bath (University of Cambridge, Cambridge, UK); Michael Flanagan (Beaumont Hospital, Dublin, Ireland); Michael Gottlieb (Rush University Medical Center, Chicago, IL, USA); Michael L. Pearl (Stony Brook University Hospital, Stony Brook, NY, USA); Michael P. Achiam (Copenhagen University Hospital Rigshospitalet, Copenhagen, Denmark); Michael Swart (Torbay Hospital, Torquay, UK); Mika Ukkonen (Tampere University Hospital, Tampere, Finland); Miklosh Bala (Hebrew University of Jerusalem, Jerusalem, Israel); Mohamed Ebrahim (Copenhagen University Hospital Amager and Hvidovre, Hvidovre, Denmark); Mohammed N. AlAli (Prince Mohammed bin Abdulaziz Hospital, Riyadh, Saudi Arabia); Monica Ortenzi (Università Politecnica delle Marche, Italy); Montassar Ghalleb (Surgical oncology department at institut Salah Azaiez, Faculté de médecine de Tunis, Université de Tunis el Manar, Tunis, Tunisia); Morten Hylander Møller (Copenhagen University Hospital - Rigshospitalet, Copenhagen, Denmark); Muhammad R. Iqbal (Norfolk and Norwich University Hospital NHS Trust, UK); Muhammed A. Ali (University Hospitals of Birmingham, Birmingham, UK); Munir Tarazi (Imperial College London, London, UK); Nicholas J. Newton (Queen Elizabeth Hospital, Birmingham, UK); Nader M. Hanna (Queen's University, Kingston, Canada); Nadia A. Henriksen (Herlev Hospital, Denmark); Natalie S. Blencowe (University of Bristol, Bristol, UK); Neil Merrett (Western Sydney University, Penrith, Australia); Neil T. Welch (Nottingham University Hospitals NHS Trust, Nottingham, UK); Nicola Colucci (Addenbrooke's CUH NHS Trust, Cambridge, UK); Nicola de'Angelis (Beaujon University Hospital (AP-HP), Paris, France); Nicola Latronico (University of Brescia, Brescia, Italy); Nicole L. Werner (University of Wisconsin School of Medicine and Public Health, Madison, WI, USA); Niels D. Martin (University of Pennsylvania, Philadelphia, PA, USA); Nikolaos Machairas (National and Kapodistrian University of Athens, Athens, Greece); Nikolay Bugaev (Tufts Medical Center, Boston, MA, USA); Ning Qi Pang (National University Health System, Singapore); Obinna Obinwa (The Royal Wolverhampton NHS Trust, Wolverhampton, UK); Onigbinde Oluwanisola Akanji (Nile University of Nigeria, Abuja, Nigeria); Panagiotis Kapsampelis (Kingston Hospital NHS Foundation Trust, London, UK); Paola De Nardi (IRCCS San Raffaele Hospital, Milan, Italy); Paolo Vincenzi (Polytechnic University of Marche, Ancona, Italy); Patricio Lamoza Kohan (SCCBM, IFSO Chile); Philip H. Pucher (Portsmouth Hospitals University NHS Trust, Portsmouth, UK); Philip J.J. Herrod (King's Mill Hospital, Mansfield, UK); Philip W.Y. CHIU (The Chinese University of Hong Kong, Hong Kong, China); Pierluigi Marzuillo (Università degli Studi della Campania “Luigi Vanvitelli”, Naples, Italy); Pierpaolo Sileri (Università Vita Salute San Raffaele, Milan, Italy); Pietro Fransvea (Fondazione Policlinico Universitario A Gemelli IRCCS, Italy); Pradeep H. Navsaria (Groote Schuur Hospital and University of Cape Town, Cape Town, South Africa); Predescu Dragos Valentin (St. Mary Clinical Hospital, Bucharest, Romania); Roel Bakx (Pediatric Surgical Center Amsterdam, Amsterdam, The Netherlands); Rachel L. Choron (Rutgers Robert Wood Johnson Medical School, New Brunswick, NJ, USA); Rahul Gupta (Synergy Institute of Medical Sciences, Uttarakhand, India); Rao R. Ivatury (Virginia Commonwealth University, Richmond, VA, USA); Raquel Diaz (University of Genova, Genova, Italy); Rebecca Anne Bradley (University Hospital Lewisham, London, UK); Reitano Elisa (Research Institute against Digestive Cancer, Strasbourg, France); René M. Palacios Huatuco (Hospital Italiano de Buenos Aires, Buenos Aires, Argentina); Reza Shahriarirad (Shiraz University of Medical Science, Shiraz, Iran); Rishi Rattan (Legacy Emanuel Medical Center, Portland, OR, USA); Riyad Karmy-Jones (Peacehealth Southwest Washington Medical Center, Vancouver, WA, USA); Robert G. Sawyer (Western Michigan University Homer Stryker MD School of Medicine, Kalamazoo, MI, USA); Robert J.S. Coelen (Dijklander Hospital, Hoorn, The Netherlands); Roberto Cirocchi (University of Perugia, Perugia, Italy); Rondi B. Gelbard (University of Alabama at Birmingham, Birmingham, AL, USA); Roxanna Zakeri (University College London Hospitals NHS Foundation Trust, London, UK); Rui Farinha (ORSI Academy, Melle, Belgium); Rutger M. Schols (Maastricht University Medical Center, Maastricht, The Netherlands); Ryan P. Dumas (University of Texas Southwestern, TX, USA); Salomone Di Saverio (Madonna del Soccorso hospital, Tronto, Italy); Samik Kumar Bandyopadhyay (Manchester Royal Infirmary, Manchester, UK); Samir Delibegovic (University Clinical Center Tuzla, Tuzla, Bosnia and Herzegovina); Sean Stevens (University of Melbourne, Heidelberg, Australia); Sergio M. Navarro (University of Minnesota, Minneapolis, MN, USA); Shamita Chatterjee (IPGME&R, Kolkata, India); Stamatios Petousis (Aristotle University of Thessaloniki, Greece); Stavros Gourgiotis (Addenbrooke's CUH NHS Trust, Cambridge, UK); Stephanie M. Streit (George Washington University, Washington DC, USA); Suman Baral (Dirghayu Pokhara Hospital Ltd, Pokhara, Nepal); Sunaina T. Karna (All India Institute of Medical Sciences, Bhopal, India); Susan Moug (Royal Alexandra Hospital, Paisley, UK); Susan Yoong (Royal Victoria Hospital, Belfast, UK); Suzanne S. Gisbertz (Amsterdam UMC location University of Amsterdam, Amsterdam, The Netherlands. Cancer Center Amsterdam, Amsterdam, the Netherlands); Tareq Kheirbek (Brown University, Providence, RI, USA); Teoh Yuen-Chun Jeremy (The Chinese University of Hong Kong, Hong Kong, China); Therese M. Duane; Thomas Korgaard Jensen (Copenhagen University Hospital Herlev-Gentofte, Copenhagen, Denmark); Tim Bright (Flinders Medical Centre, Bedford Park, Australia); Timothy Craig Hardcastle (University of KwaZulu-Natal and Inkosi Albert Luthuli Central Hospital, Durban, South Africa); Triantafyllou Tania (Royal Infirmary of Edinburgh, Edinburgh, UK); Vahagn C. Nikolian (Oregon Health & Science University, Portland, OR, USA); Valentina Bianchi (Fondazione Policlinico Universitario A Gemelli IRCCS, Italy); Victor Kong (University of KwaZulu Natal, Durban, South Africa); Vincenzo Trapani (Baggiovara General Hospital, Modena, Italy); Vishal G. Shelat (Tan Tock Seng Hospital, Singapore); Vishnu R. Mani (The University of Iowa Hospitals and Clinics, Iowa City, IA, USA); Vladimir M.Khokha (Belarus Association of Surgeons, Belarus); Wah Yang (The First Affiliated Hospital of Jinan University, Guangzhou, China); Waleed Al-Khyatt (Royal Derby Hospital, Derby, UK); Yick Ho Lam (Lyell McEwin Hospital, Adelaide, Australia); Yu Kijima (Tokyo Women's Medical University, Tokyo, Japan); Yunfeng Cui (Tianjin Nankai Hospital, Tianjin, China); Zane B. Perkins (Royal London Hospital, London, UK); Zaza Demetrashvili (Tbilisi State Medical University, Georgia); Zi Qin Ng (Royal Perth Hospital, Perth, Australia).

## Supplementary Material

zrad145_Supplementary_Data

## Data Availability

All data for the present study is either included as supplementary material or obtainable upon reasonable request from the authors.
