# Supplementary material for: Novel Textbook Outcomes following emergency laparotomy: Delphi exercise
Source: BJS Open. 2024 Jan 29;8(1):zrad145. doi: 10.1093/bjsopen/zrad145 (PMC10823418; doi:10.1093/bjsopen/zrad145)
Supplement: zrad145_Supplementary_Data [file zrad145_supplementary_data.pdf]

# Novel Textbook Outcomes following emergency laparotomy: a Delphi exercise


**Authors**

David N Naumann[a], Aneel Bhangu[a,b], Adam Brooks[c], Matthew Martin[d], Bryan A Cotton[e], Mansoor Khan[f], Mark J Midwinter[g], Lyndsay Pearce[h], Douglas M Bowley[a], John B Holcomb[i], Ewen A Griffiths[a], and the BEACON Collaborative[j]

**Affiliations**

[a]Department of Trauma and Emergency General Surgery, University Hospitals Birmingham NHS Foundation Trust, Birmingham, UK

[b]NIHR Global Health Unit on Global Surgery, Institute of Translational Medicine, University of Birmingham, Birmingham, UK

[c]East Midlands Major Trauma Centre, Queen's Medical Centre, Nottingham, UK

[d]Division of Trauma and Acute Care Surgery, Dept. of Surgery, Los Angeles County & USC Medical Center, Los Angeles, CA

[e]The Center for Translational Injury Research, McGovern Medical School at The University of Texas Health Science Center at Houston, Houston, Texas

[f]University Hospitals Sussex NHS Foundation Trust, Brighton, UK

[g]School of Biomedical Sciences, The University of Queensland, Brisbane, Australia

[h]Salford Royal NHS Foundation Trust, Salford, UK

[i]Division of Acute Care Surgery, Department of Surgery, University of Alabama at Birmingham, Birmingham, USA

[j]Names and affiliations listed in the Collaborators section

**Corresponding author**

Dr David N Naumann PhD FRCS, Department of Trauma and Emergency General Surgery, University Hospitals Birmingham NHS Foundation Trust, Mindlesohn Way, Birmingham, B15 2TH, United Kingdom

Email: d.n.naumann@bham.ac.uk

Tel: +44 (0) 7861 242 807

Twitter/X: @DavidNNaumann

ORCID ID: 0000-0003-2243-2325

## Supplementary Materials - Index

### Supplementary Results


Delphi Round 1. Delphi Round 1 *pages 3 - 14*

Delphi Round 2. Delphi Round 2 *pages 15 - 48*

Delphi Round 3. Delphi Round 3 *pages 49 - 53*


### Supplementary Figures and Tables


Full list of countries of participants *pages 54 - 55*

## Supplementary Results 1. Delphi Round 1



# Textbook Outcomes for patients following emergency laparotomy

Please complete the survey below.

Thank you!

---

1) Please read the following: What are Textbook Outcomes? "Textbook Outcomes" are composite measures that incorporate multiple patient outcomes representing the "ideal" or best possible outcome. Rather than an individual outcome such as "survival" after cancer resection, an example of a Textbook Outcome may be "returned home alive and without any surgical complications, with a R0 resection and appropriately radical lymphadenectomy on histology".

What is the purpose of this Delphi Exercise? This Delphi exercise seeks to define which Textbook Outcomes might be the best to use for patients following emergency laparotomy, based on your expert opinion[1].

What will be done with my responses? Your anonymous, voluntary responses to this round of questions will be used to inform the next round of survey questions in order to achieve consensus amongst experts in their field.

References 1. Naumann DN, Bhangu A, Brooks A, Martin M, Cotton BA, Khan M, Midwinter MJ, Pearce L, Bowley DM, Holcomb JB, Griffiths EA. A call for patient-centred textbook outcomes for emergency surgery and trauma. Br J Surg. 2022 (https://academic.oup.com/bjs/advance-article/doi/10.1093/bjs/znac271/6670913?searchresult=1)

◯ I understand all of this and wish to proceed with the survey

---

2) What is your current role in emergency surgery and trauma?

◯ I am an Attending or Consultant grade doctor
◯ I am a Non-Attending or non-Consultant grade of doctor (e.g. trainee / junior doctor)
◯ I am not a medical doctor (insert role below)
◯ I am a researcher as my primary role
◯ I am a patient

---

3) If you are not a medically trained doctor, please write your primary role

__________________________________

4) In what year did you graduate from medical school? (leave blank if you did not attend medical school)

- ◯ 1975
- ◯ 1976
- ◯ 1977
- ◯ 1978
- ◯ 1979
- ◯ 1980
- ◯ 1981
- ◯ 1982
- ◯ 1983
- ◯ 1984
- ◯ 1985
- ◯ 1986
- ◯ 1987
- ◯ 1988
- ◯ 1989
- ◯ 1990
- ◯ 1991
- ◯ 1992
- ◯ 1993
- ◯ 1994
- ◯ 1995
- ◯ 1996
- ◯ 1997
- ◯ 1998
- ◯ 1999
- ◯ 2000
- ◯ 2001
- ◯ 2002
- ◯ 2003
- ◯ 2004
- ◯ 2005
- ◯ 2006
- ◯ 2007
- ◯ 2008
- ◯ 2009
- ◯ 2010
- ◯ 2011
- ◯ 2012
- ◯ 2013
- ◯ 2014
- ◯ 2015
- ◯ 2016
- ◯ 2017
- ◯ 2018
- ◯ 2019
- ◯ 2020

5) What country do you work in for most of your time?

- ◯ Afghanistan
- ◯ Albania
- ◯ Algeria
- ◯ Andorra
- ◯ Angola
- ◯ Antigua & Deps
- ◯ Argentina
- ◯ Armenia
- ◯ Australia
- ◯ Austria
- ◯ Azerbaijan
- ◯ Bahamas
- ◯ Bahrain
- ◯ Bangladesh
- ◯ Barbados
- ◯ Belarus
- ◯ Belgium
- ◯ Belize
- ◯ Benin
- ◯ Bhutan
- ◯ Bolivia
- ◯ Bosnia Herzegovina
- ◯ Botswana
- ◯ Brazil
- ◯ Brunei
- ◯ Bulgaria
- ◯ Burkina
- ◯ Burundi
- ◯ Cambodia
- ◯ Cameroon
- ◯ Canada
- ◯ Cape Verde
- ◯ Central African Rep
- ◯ Chad
- ◯ Chile
- ◯ China
- ◯ Colombia
- ◯ Comoros
- ◯ Congo
- ◯ Congo {Democratic Rep}
- ◯ Costa Rica
- ◯ Croatia
- ◯ Cuba
- ◯ Cyprus
- ◯ Czech Republic
- ◯ Denmark
- ◯ Djibouti
- ◯ Dominica
- ◯ Dominican Republic
- ◯ East Timor
- ◯ Ecuador
- ◯ Egypt
- ◯ El Salvador
- ◯ Equatorial Guinea
- ◯ Eritrea
- ◯ Estonia
- ◯ Ethiopia
- ◯ Fiji
- ◯ Finland
- ◯ France
- ◯ Gabon
- ◯ Gambia
- ◯ Georgia
- ◯ Germany
- ◯ Ghana
- ◯ Greece
- ◯ Grenada
- ◯ Guatemala

◯ Guinea
◯ Guinea-Bissau
◯ Guyana
◯ Haiti
◯ Honduras
◯ Hungary
◯ Iceland
◯ India
◯ Indonesia
◯ Iran
◯ Iraq
◯ Ireland {Republic}
◯ Israel
◯ Italy
◯ Ivory Coast
◯ Jamaica
◯ Japan
◯ Jordan
◯ Kazakhstan
◯ Kenya
◯ Kiribati
◯ Korea North
◯ Korea South
◯ Kosovo
◯ Kuwait
◯ Kyrgyzstan
◯ Laos
◯ Latvia
◯ Lebanon
◯ Lesotho
◯ Liberia
◯ Libya
◯ Liechtenstein
◯ Lithuania
◯ Luxembourg
◯ Macedonia
◯ Madagascar
◯ Malawi
◯ Malaysia
◯ Maldives
◯ Mali
◯ Malta
◯ Marshall Islands
◯ Mauritania
◯ Mauritius
◯ Mexico
◯ Micronesia
◯ Moldova
◯ Monaco
◯ Mongolia
◯ Montenegro
◯ Morocco
◯ Mozambique
◯ {Burma}
◯ Namibia
◯ Nauru
◯ Nepal
◯ Netherlands
◯ New Zealand
◯ Nicaragua
◯ Niger
◯ Nigeria
◯ Norway
◯ Oman
◯ Pakistan
◯ Palau
◯ Panama
◯ Papua New Guinea
◯ Paraguay
◯ Peru
◯ Philippines

◯ Poland
◯ Portugal
◯ Qatar
◯ Romania
◯ Russian Federation
◯ Rwanda
◯ St Kitts & Nevis
◯ St Lucia
◯ Saint Vincent & the Grenadines
◯ Samoa
◯ San Marino
◯ Sao Tome & Principe
◯ Saudi Arabia
◯ Senegal
◯ Serbia
◯ Seychelles
◯ Sierra Leone
◯ Singapore
◯ Slovakia
◯ Slovenia
◯ Solomon Islands
◯ Somalia
◯ South Africa
◯ South Sudan
◯ Spain
◯ Sri Lanka
◯ Sudan
◯ Suriname
◯ Swaziland
◯ Sweden
◯ Switzerland
◯ Syria
◯ Taiwan
◯ Tajikistan
◯ Tanzania
◯ Thailand
◯ Togo
◯ Tonga
◯ Trinidad & Tobago
◯ Tunisia
◯ Turkey
◯ Turkmenistan
◯ Tuvalu
◯ Uganda
◯ Ukraine
◯ United Arab Emirates
◯ United Kingdom
◯ United States
◯ Uruguay
◯ Uzbekistan
◯ Vanuatu
◯ Vatican City
◯ Venezuela
◯ Vietnam
◯ Yemen
◯ Zambia
◯ Zimbabwe

---

6) What is your gender?

◯ Female
◯ Male
◯ Other
◯ I'd prefer not to say

7) Do you regularly perform NON-TRAUMA emergency laparotomy and/or regularly look after patients who have had an emergency laparotomy (NOT FOR TRAUMA)?

- ◯ Yes
- ◯ No

8) Do you regularly perform emergency TRAUMA laparotomy and/or regularly look after patients who have had an emergency TRAUMA laparotomy?

- ◯ Yes
- ◯ No

9) Approximately how many emergency NON-TRAUMA laparotomies have you performed as the first surgeon?

- ◯ None
- ◯ < 100
- ◯ 100-300
- ◯ >300

10) Approximately how many emergency TRAUMA laparotomies have you performed as the first surgeon?

- ◯ None
- ◯ < 100
- ◯ 100-300
- ◯ >300

11) Delphi Q1.
Do you think that the use of Textbook Outcomes for patients following emergency laparotomy (for both trauma and non-trauma indications) may be of some value*?

*please refer to the top of the page for definition of Textbook Outcome

- ◯ Yes
- ◯ No

Dephi Q2. Please answer the following questions according to a Likert scale of 1 - 5:

Example:
Strongly agree
Agree
Neutral
Disagree
Strongly disagree

Please leave any comments in the free text section below (Delphi Q5)

12) Q2.1.a. "Mortality/survival" should be incorporated into the Textbook Outcome for emergency NON-TRAUMA laparotomy

Stronly disagree Neutral Strongly agree

_(Place a mark on the scale above)_

13) Q2.1.b. "Mortality/survival" should be incorporated into the Textbook Outcome for emergency TRAUMA laparotomy

Stronly disagree Neutral Strongly agree

_(Place a mark on the scale above)_

14) Q2.2.a. "Time to normalise lactate" should be incorporated into the Textbook Outcome for emergency NON-TRAUMA laparotomy

Stronly disagree Neutral Strongly agree

_(Place a mark on the scale above)_

15) Q2.2.b. "Time to normalise lactate" should be incorporated into the Textbook Outcome for emergency TRAUMA laparotomy

Stronly disagree Neutral Strongly agree

_(Place a mark on the scale above)_

16) Q2.3.a. "Blood products used" should be incorporated into the Textbook Outcome for emergency NON-TRAUMA laparotomy

Stronly disagree Neutral Strongly agree

_(Place a mark on the scale above)_

17) Q2.3.b. "Blood products used" should be incorporated into the Textbook Outcome for emergency TRAUMA laparotomy

Stronly disagree Neutral Strongly agree

_(Place a mark on the scale above)_

18) Q2.4.a. "Fascial closure" (excluding vicryl mesh closure) should be incorporated into the Textbook Outcome for emergency NON-TRAUMA laparotomy

Stronly disagree Neutral Strongly agree

_(Place a mark on the scale above)_

19) Q2.4.b. "Fascial closure" (excluding vicryl mesh closure) should be incorporated into the Textbook Outcome for emergency TRAUMA laparotomy

Stronly disagree Neutral Strongly agree

_(Place a mark on the scale above)_

20) Q2.5.a "Overall post-operative complications" should be incorporated into the Textbook Outcome for emergency NON-TRAUMA laparotomy

Stronly disagree Neutral Strongly agree

_(Place a mark on the scale above)_

21) Q2.5.b "Overall post-operative complications" should be incorporated into the Textbook Outcome for emergency TRAUMA laparotomy

Stronly disagree Neutral Strongly agree

_(Place a mark on the scale above)_

22) Q2.6.a. "Post-operative intra-abdominal sepsis / anastomotic leak / abscess / fistula" should be incorporated into the Textbook Outcome for emergency NON-TRAUMA laparotomy

Stronly disagree Neutral Strongly agree

_(Place a mark on the scale above)_

23) Q2.6.b. "Post-operative intra-abdominal sepsis / anastomotic leak / abscess / fistula" should be incorporated into the Textbook Outcome for emergency TRAUMA laparotomy

Stronly disagree Neutral Strongly agree

_(Place a mark on the scale above)_

24) Q2.7.a. "Organ failure (or individual organ failures)" should be incorporated into the Textbook Outcome for emergency NON-TRAUMA laparotomy

Stronly disagree | Neutral | Strongly agree

*(Place a mark on the scale above)*

25) Q2.7.b. "Organ failure (or individual organ failures)" should be incorporated into the Textbook Outcome for emergency TRAUMA laparotomy

Stronly disagree | Neutral | Strongly agree

*(Place a mark on the scale above)*

26) Q2.8.a. "Venous thromboembolism (DVT or PE)" should be incorporated into the Textbook Outcome for emergency NON-TRAUMA laparotomy

Stronly disagree | Neutral | Strongly agree

*(Place a mark on the scale above)*

27) Q2.8.b. "Venous thromboembolism (DVT or PE)" should be incorporated into the Textbook Outcome for emergency TRAUMA laparotomy

Stronly disagree | Neutral | Strongly agree

*(Place a mark on the scale above)*

28) Q2.9.a. "Unplanned re-operation" should be incorporated into the Textbook Outcome for emergency NON-TRAUMA laparotomy

Stronly disagree | Neutral | Strongly agree

*(Place a mark on the scale above)*

29) Q2.9.b. "Unplanned re-operation" should be incorporated into the Textbook Outcome for emergency TRAUMA laparotomy

Stronly disagree | Neutral | Strongly agree

*(Place a mark on the scale above)*

30) Q2.10.a. "Post-operative hernia" should be incorporated into the Textbook Outcome for emergency NON-TRAUMA laparotomy

Stronly disagree | Neutral | Strongly agree

*(Place a mark on the scale above)*

31) Q2.10.b. "Post-operative hernia" should be incorporated into the Textbook Outcome for emergency TRAUMA laparotomy

Stronly disagree | Neutral | Strongly agree

*(Place a mark on the scale above)*

32) Q2.11.a. "Length of stay in hospital" should be incorporated into the Textbook Outcome for emergency NON-TRAUMA laparotomy

Stronly disagree | Neutral | Strongly agree

*(Place a mark on the scale above)*

33) Q2.11.b. "Length of stay in hospital" should be incorporated into the Textbook Outcome for emergency TRAUMA laparotomy

Stronly disagree | Neutral | Strongly agree

*(Place a mark on the scale above)*

34) Q2.12.a. "Length of stay in the Intensive Care Unit" should be incorporated into the Textbook Outcome for emergency NON-TRAUMA laparotomy

Stronly disagree Neutral Strongly agree

*(Place a mark on the scale above)*

35) Q2.12.b "Length of stay in the Intensive Care Unit" should be incorporated into the Textbook Outcome for emergency TRAUMA laparotomy

Stronly disagree Neutral Strongly agree

*(Place a mark on the scale above)*

36) Q2.13.a. A form of functional or quality of life outcome should be incorporated into the Textbook Outcome for emergency NON-TRAUMA laparotomy

Stronly disagree Neutral Strongly agree

*(Place a mark on the scale above)*

37) Q2.13.b. A form of functional or quality of life outcome should be incorporated into the Textbook Outcome for emergency TRAUMA laparotomy

Stronly disagree Neutral Strongly agree

*(Place a mark on the scale above)*

38) Q2.14.a. Requirement for ongoing rehabilitation should be incorporated into the Textbook Outcome for emergency NON-TRAUMA laparotomy

Stronly disagree Neutral Strongly agree

*(Place a mark on the scale above)*

39) Q2.14.b. Requirement for ongoing rehabilitation should be incorporated into the Textbook Outcome for emergency TRAUMA laparotomy

Stronly disagree Neutral Strongly agree

*(Place a mark on the scale above)*

40) Q2.15.a. "Return to work" should be incorporated into the Textbook Outcome for emergency NON-TRAUMA laparotomy

Stronly disagree Neutral Strongly agree

*(Place a mark on the scale above)*

41) Q2.15.b. "Return to work" should be incorporated into the Textbook Outcome for emergency TRAUMA laparotomy

Stronly disagree Neutral Strongly agree

*(Place a mark on the scale above)*

42) Q2.16. Follow up period should be:

◯ 24 hours ◯ 30 days
◯ 60 days ◯ 90 days
◯ 6 months ◯ 1 year
◯ Other: I will leave a comment below in Q4

Please have a look at this Table before answering the next question

**Table 1 Eligibility criteria for textbook outcomes in emergency surgery and trauma**

| Criterion | Domain | Statement |
|---|---|---|
| 1 | Non-intrusive | Measurable without undue distress or inconvenience to patients |
| 2 | Realistic | Achievable and realistic according to the initial patient presentation |
| 3 | Relevant | Relevant to patients, and updated/reviewed at regular intervals |
| 4 | Consensus | Agreed through consensus opinion and mutual agreement between healthcare providers, patients, and the public |
| 5 | Clarity | Easy to understand, without overly complicated or confusing components |

43) Delphi Q3. How many individual outcomes do you believe can realistically be combined in a composite Textbook Outcome to be practical and useful?

◯ 2 ◯ 3 ◯ 4 ◯ 5
◯ 6 ◯ 7 ◯ 8 ◯ 9
◯ 10 or more

44) Delphi Q4. Patients would agree with the decisions I have made here

Stronly disagree — Neutral — Strongly agree

*(Place a mark on the scale above)*

45) Delphi Q5.

Please insert your own comments in free text regarding individual outcome measures that you think should be incorporated into the Textbook Outcome for energency laparotomy. Or, you may also wish to propose a Textbook Outcome for emergency laparotomy in full, incorporating all of the individual components that you feel are most important.

As a prompt, here are some individual outcome measures proposed as important in a recent study of trauma laparotomy [Byerly S et al. A core outcome set for damage control laparotomy via modified Delphi method. Trauma Surgery & Acute Care Open. 2022;7(1):e000821-e].

Blood product transfusion (whole blood vs component transfusion)

24-hour packed red blood cells

Time to normalize lactate

Time to hemostasis (surgical control of bleeding)

Time interval between operations

Involvement of specialty surgeon at second operation

Time to enteral feeding

Fascial closure (vicryl mesh is NOT a fascial closure) at index hospitalization

Abdominal closure

Days to fascial closure

(Planned) ventral hernia

Postoperative ventral hernia formation 12–24 months follow-up

90-day postoperative complication rate

Complications/Abdominal complications

Major complications requiring reoperations/unplanned re-exploration following closure

Sepsis/Intra-abdominal sepsis

Intra-abdominal abscess/surgical site infection III/deep space infection

Gastrointestinal anastomotic leak, if applicable

Secondary intra-abdominal sepsis (including anastomotic leak)

Enterocutaneous fistula

Acute kidney injury/presence and degree of organ failure

Acute respiratory distress syndrome

Deep vein thrombosis

Hospital length of stay

Intensive care unit length of stay

Ventilator days

Intensive care unity length of stay with ventilator free days

Hospital mortality

28-day mortality

30-day mortality

90-day mortality (not sooner)

Long-term physical and psychological functional assessment

Functional outcome at 12 months (return to work, pain score, etc)

---

46) Do you wish to make any further comments about this survey, or ways in which it might be imporved in future rounds of the Delphi exercise? ____________________

---

47) Would you like to participate in the full Delphi exercise entitled: "Textbook Outcomes following emergency laparotomy"? This will include at least 2 and up to 4 rounds of questions.

◯ Yes
◯ No

---

48) Please add your email address that is to be used for subsequent rounds of this Delphi excercise: ____________________

---

49) If you would like to be listed as a co-author, please insert your ORCID ID here: ____________________

Link to ORCID: https://orcid.org/

**Supplementary Results 2. Delphi Round 2**



# ROUND 2 of Delphi - Textbook Outcomes following emergency laparotomy

Please complete the survey below.

Thank you!

1) Round 2 of the Delphi process:

Textbook Outcomes for Trauma and Emergency General Surgery

You are one of 411 participants from Round 1. Please complete Round 2 in order to be eligible for authorship in the final publication. Reminder: 1. What are Textbook Outcomes? "Textbook Outcomes" are composite measures that incorporate multiple patient outcomes representing the “ideal” or best possible outcome. Rather than an individual outcome such as “survival” after cancer resection, an example of a Textbook Outcome may be “returned home alive and without any surgical complications, with a R0 resection and appropriately radical lymphadenectomy on histology”.

2. What is the purpose of this Delphi Exercise? This Delphi exercise seeks to define which Textbook Outcomes might be the best to use for patients following emergency laparotomy, based on your expert opinion[1].

3. What will be done with my responses? Your anonymous, voluntary responses to this second round of questions will be used to inform the final round of survey questions in order to achieve consensus amongst experts in their field.

4. References 1. Naumann DN, Bhangu A, Brooks A, Martin M, Cotton BA, Khan M, Midwinter MJ, Pearce L, Bowley DM, Holcomb JB, Griffiths EA. A call for patient-centred textbook outcomes for emergency surgery and trauma. Br J Surg. 2022 (https://academic.oup.com/bjs/advance-article/doi/10.1093/bjs/znac271/6670913?searchresult=1)

○ I understand all of this and wish to proceed with the survey

2) Please confirm your email address here, so that we can confirm your participation and prepare for the third (final) round of the Delphi

__________________________________

Each question below has the Round 1 results displayed as a bar-chart, with the red bar indicating the most popular score.

Please answer the questions with the Likert Scale 1-5 as before, taking into account the opinions from your colleagues in Round 1

RESULTS FROM ROUND 1: "Mortality/survival" should be incorporated into the Textbook Outcome for emergency NON-TRAUMA laparotomy

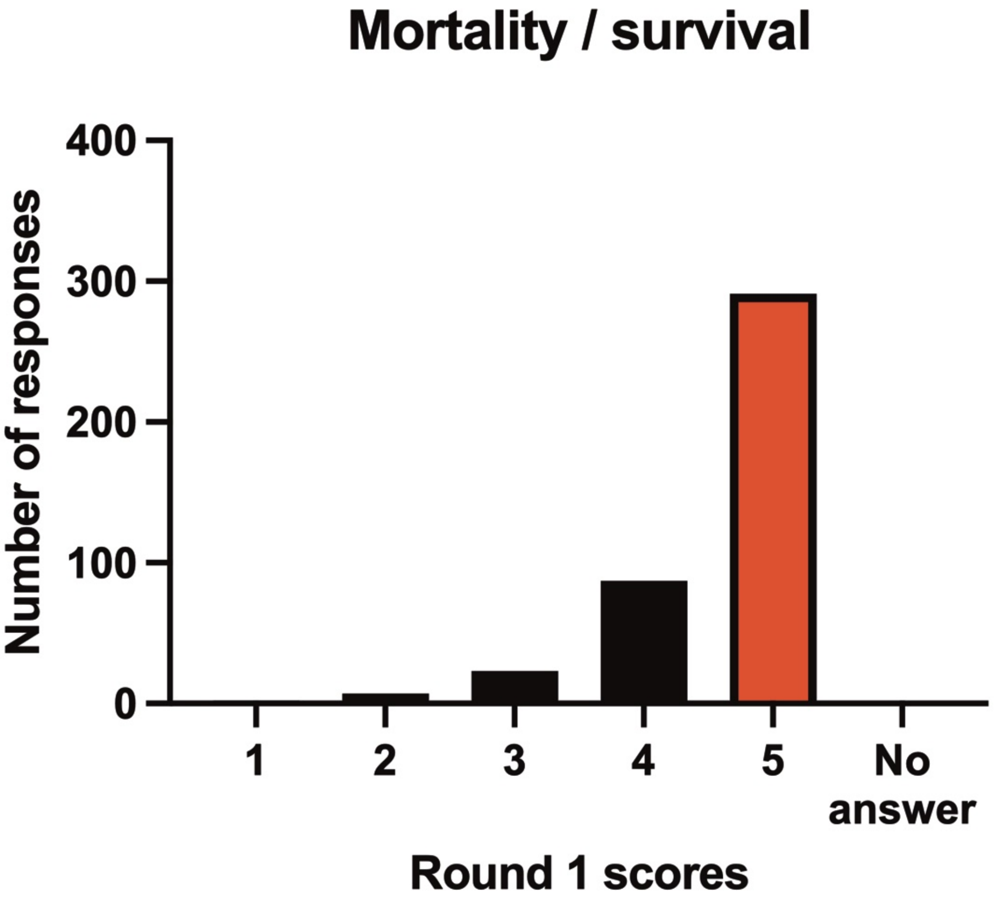


3) Your new answer: "Mortality/survival" should be incorporated into the Textbook Outcome for emergency NON-TRAUMA laparotomy

Strongly disagree | Neutral | Strongly agree

*(Place a mark on the scale above)*

RESULTS FROM ROUND 1 "Mortality/survival" should be incorporated into the Textbook Outcome for emergency TRAUMA laparotomy

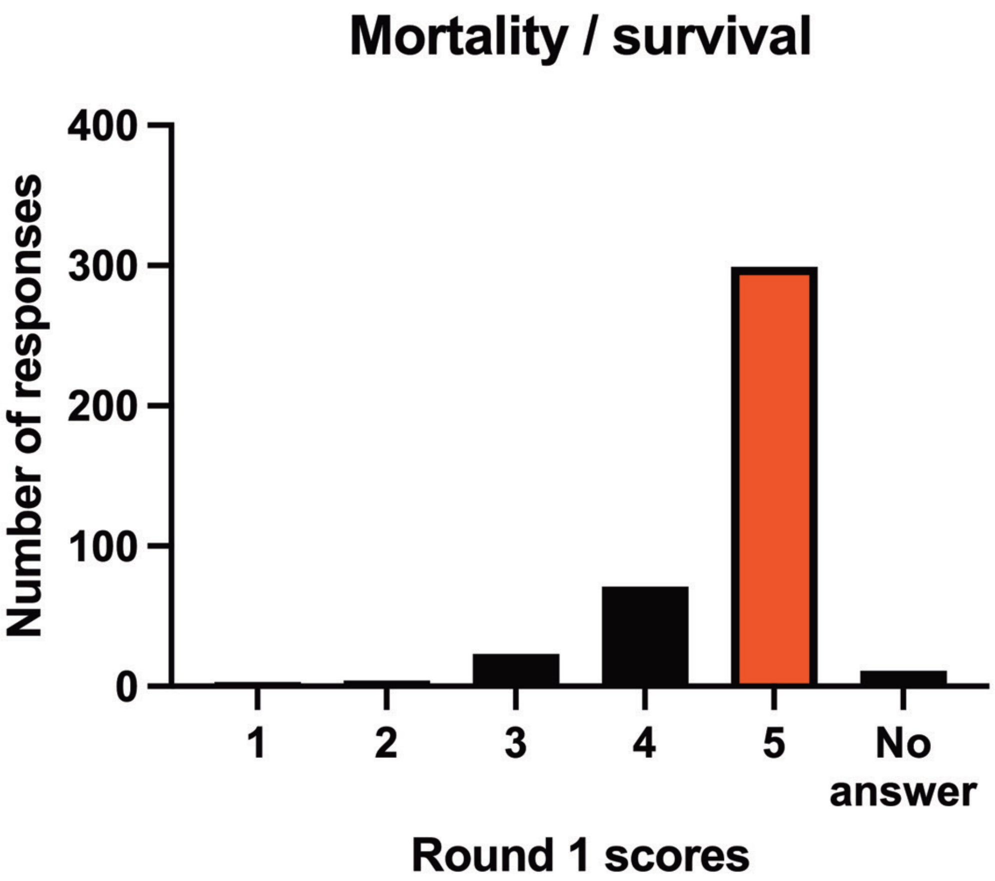


4) Your new answer: "Mortality/survival" should be incorporated into the Textbook Outcome for emergency TRAUMA laparotomy

Strongly disagree Neutral Strongly agree

_(Place a mark on the scale above)_

RESULTS FROM ROUND 1 "Time to normalise lactate" should be incorporated into the Textbook Outcome for emergency NON-TRAUMA laparotomy

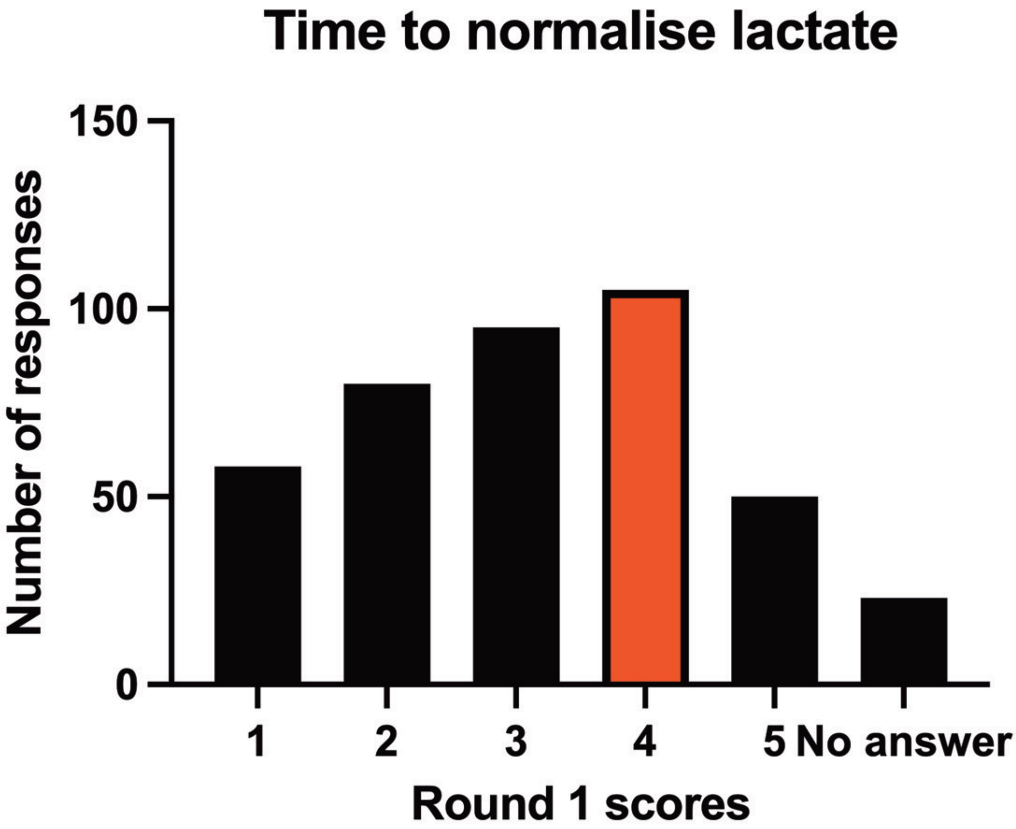


5) Your new answer: "Time to normalise lactate" should be incorporated into the Textbook Outcome for emergency NON-TRAUMA laparotomy

Strongly disagree Neutral Strongly agree

*(Place a mark on the scale above)*

RESULTS FROM ROUND 1 "Time to normalise lactate" should be incorporated into the Textbook Outcome for emergency TRAUMA laparotomy

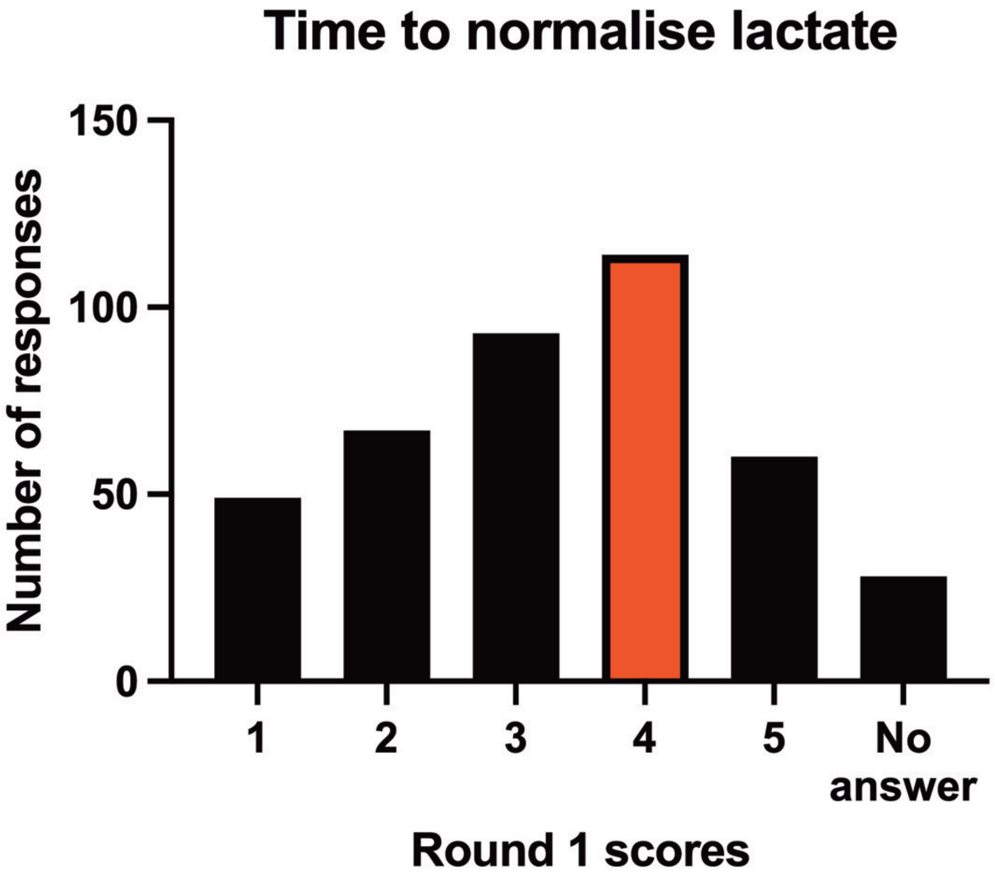


6) Your new answer: "Time to normalise lactate" should be incorporated into the Textbook Outcome for emergency TRAUMA laparotomy

Strongly disagree Neutral Strongly agree

*(Place a mark on the scale above)*

RESULTS FROM ROUND 1 "Blood products used" should be incorporated into the Textbook Outcome for emergency NON-TRAUMA laparotomy

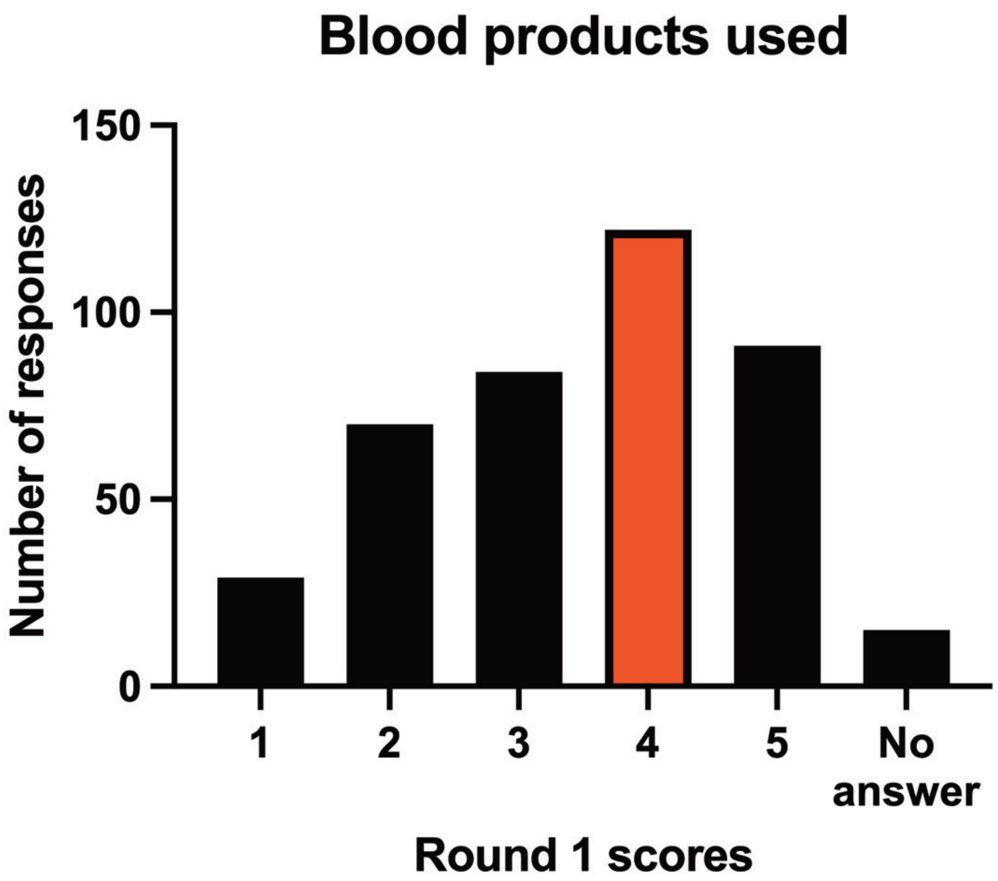


7) Your new answer: "Blood products used" should be incorporated into the Textbook Outcome for emergency NON-TRAUMA laparotomy

Strongly disagree Neutral Strongly agree

*(Place a mark on the scale above)*

RESULTS FROM ROUND 1 "Blood products used" should be incorporated into the Textbook Outcome for emergency TRAUMA laparotomy

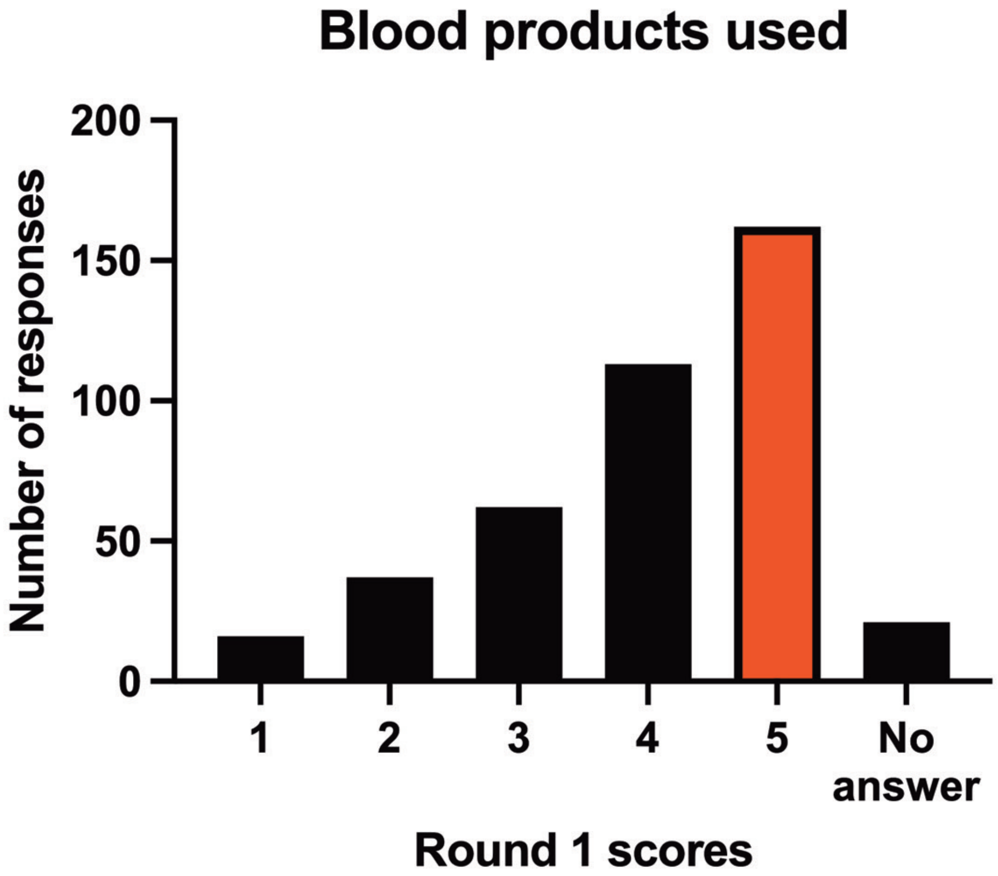


8) Your new answer: "Blood products used" should be incorporated into the Textbook Outcome for emergency TRAUMA laparotomy

Strongly disagree Neutral Strongly agree

*(Place a mark on the scale above)*

RESULTS FROM ROUND 1 "Fascial closure" (excluding vicryl mesh closure) should be incorporated into the Textbook Outcome for emergency NON-TRAUMA laparotomy

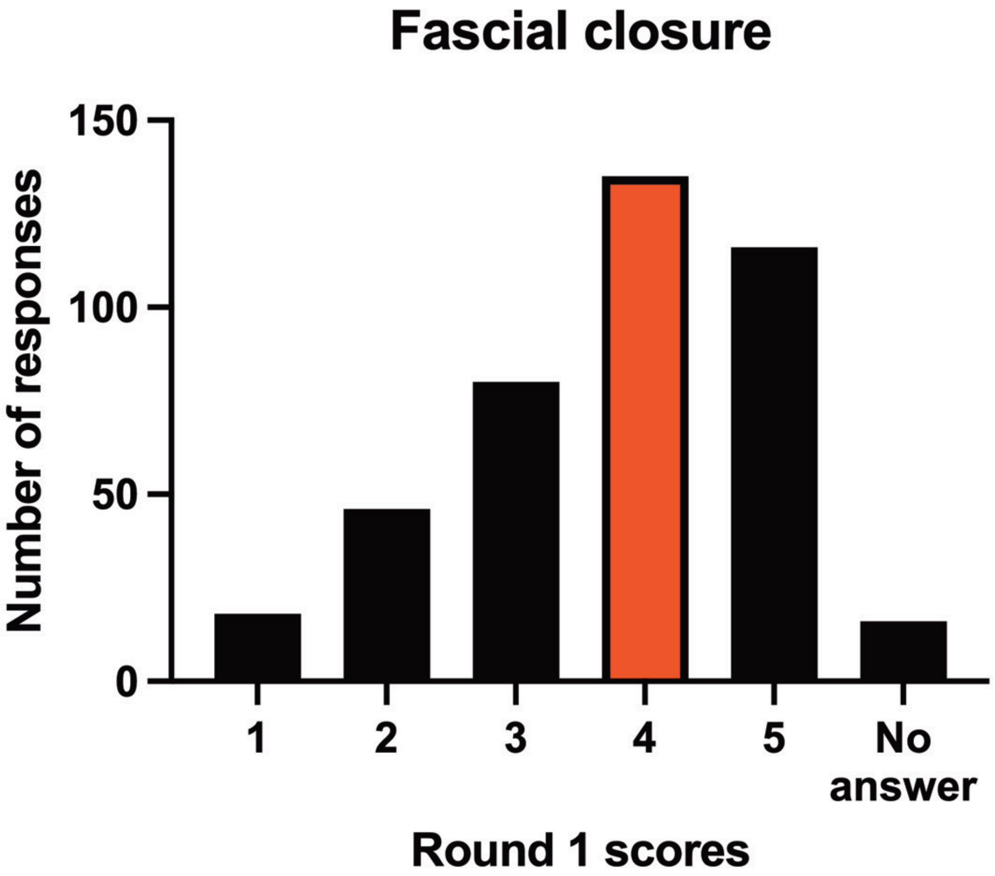


9) Your new answer: "Fascial closure" (excluding vicryl mesh closure) should be incorporated into the Textbook Outcome for emergency NON-TRAUMA laparotomy

Strongly disagree Neutral Strongly agree

*(Place a mark on the scale above)*

RESULTS FROM ROUND 1 "Fascial closure" (excluding vicryl mesh closure) should be incorporated into the Textbook Outcome for emergency TRAUMA laparotomy

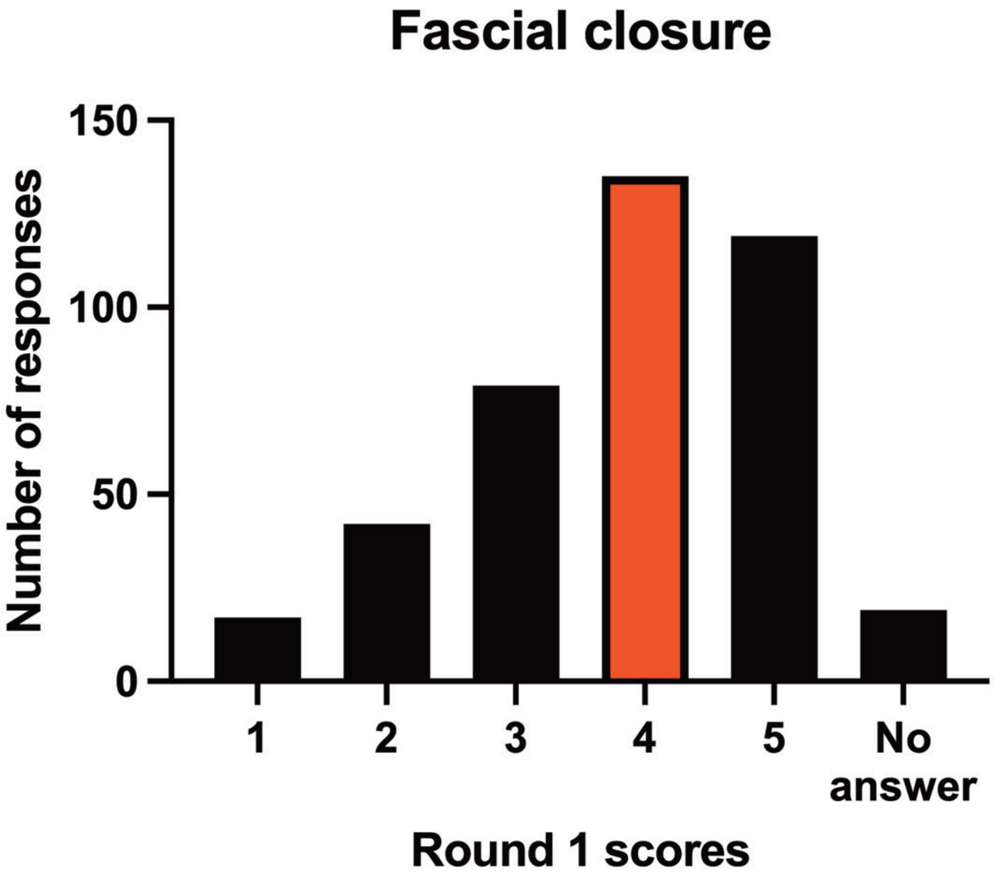


10) Your new answer: "Fascial closure" (excluding vicryl mesh closure) should be incorporated into the Textbook Outcome for emergency TRAUMA laparotomy

Strongly disagree | Neutral | Strongly agree

_(Place a mark on the scale above)_

RESULTS FROM ROUND 1 "Overall post-operative complications" should be incorporated into the Textbook Outcome for emergency NON-TRAUMA laparotomy

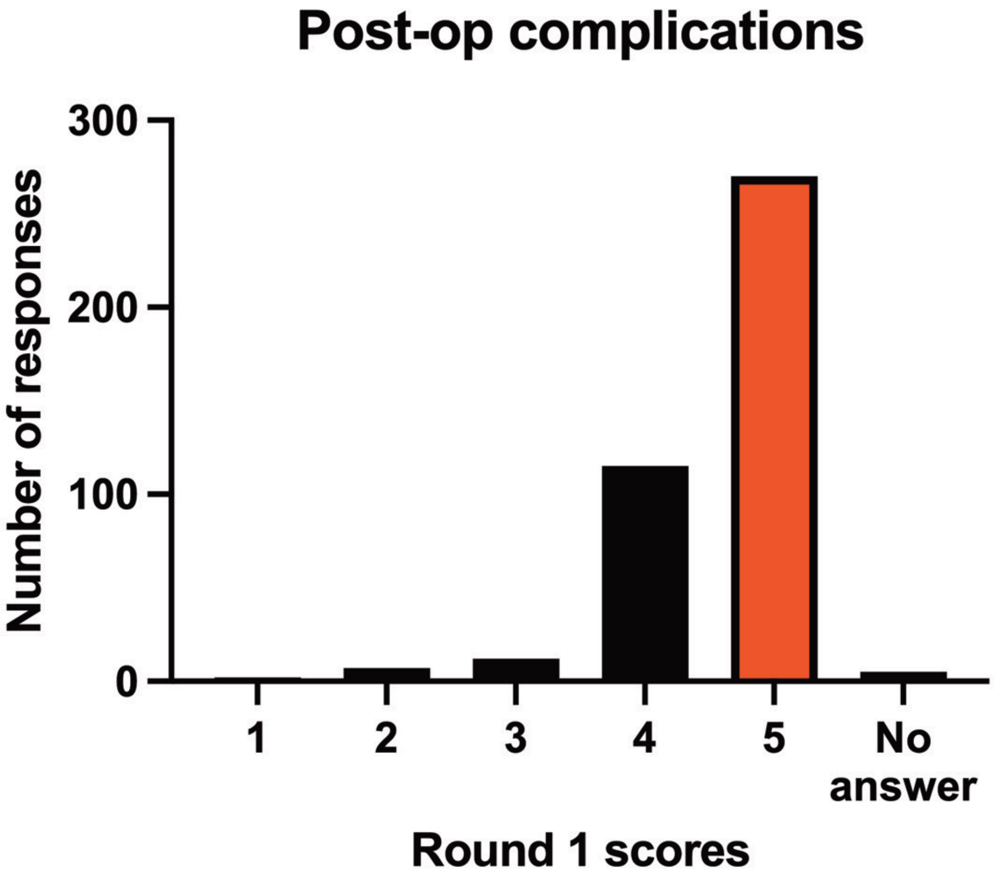


11) Your new answer: "Overall post-operative complications" should be incorporated into the Textbook Outcome for emergency NON-TRAUMA laparotomy

Strongly disagree | Neutral | Strongly agree

*(Place a mark on the scale above)*

RESULTS FROM ROUND 1 "Overall post-operative complications" should be incorporated into the Textbook Outcome for emergency TRAUMA laparotomy

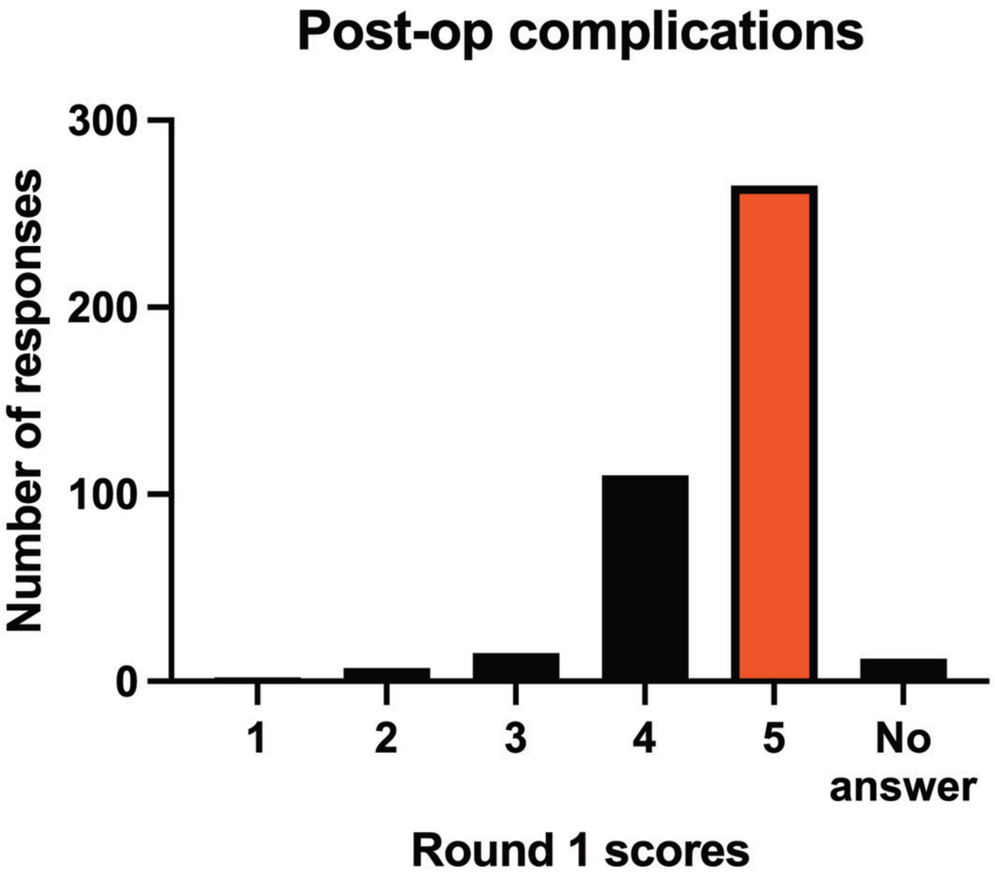


12) Your new answer: "Overall post-operative complications" should be incorporated into the Textbook Outcome for emergency TRAUMA laparotomy

Strongly disagree  Neutral  Strongly agree

*(Place a mark on the scale above)*

RESULTS FROM ROUND 1 "Post-operative intra-abdominal sepsis / anastomotic leak / abscess / fistula" should be incorporated into the Textbook Outcome for emergency NON-TRAUMA laparotomy

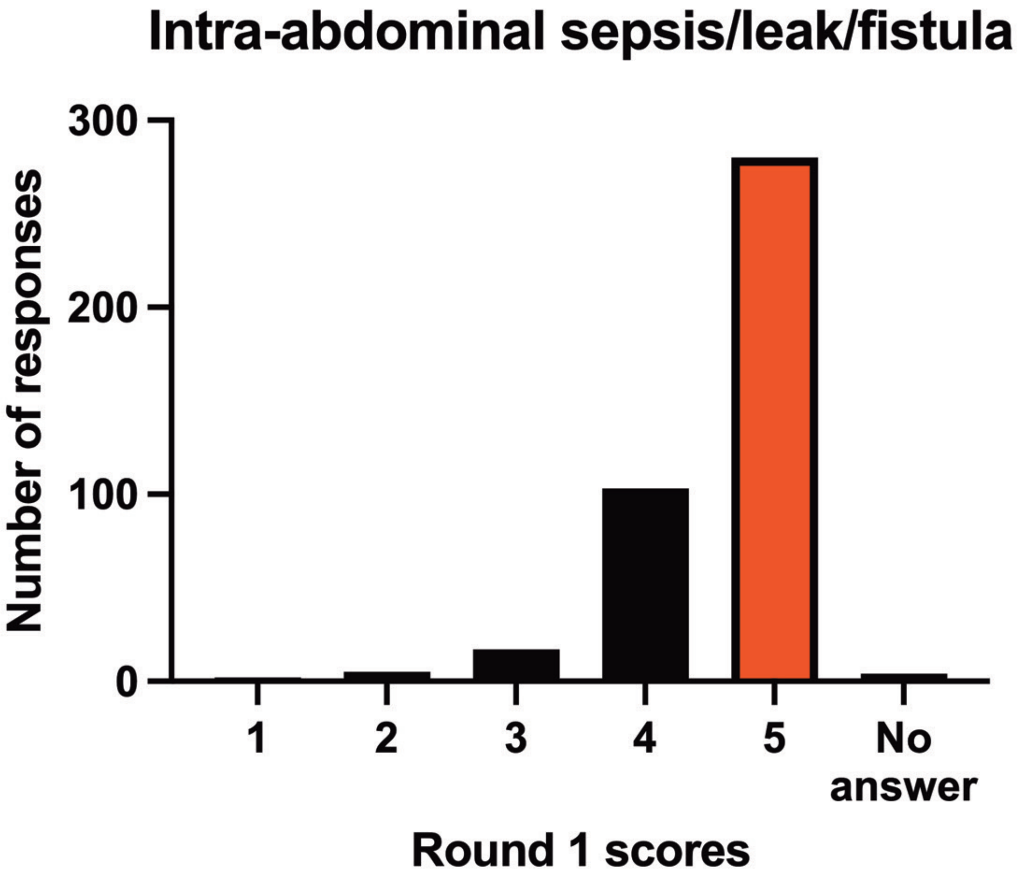


13) Your new answer: "Post-operative intra-abdominal sepsis / anastomotic leak / abscess / fistula" should be incorporated into the Textbook Outcome for emergency NON-TRAUMA laparotomy

Strongly disagree — Neutral — Strongly agree

*(Place a mark on the scale above)*

RESULTS FROM ROUND 1 "Post-operative intra-abdominal sepsis / anastomotic leak / abscess / fistula" should be incorporated into the Textbook Outcome for emergency TRAUMA laparotomy

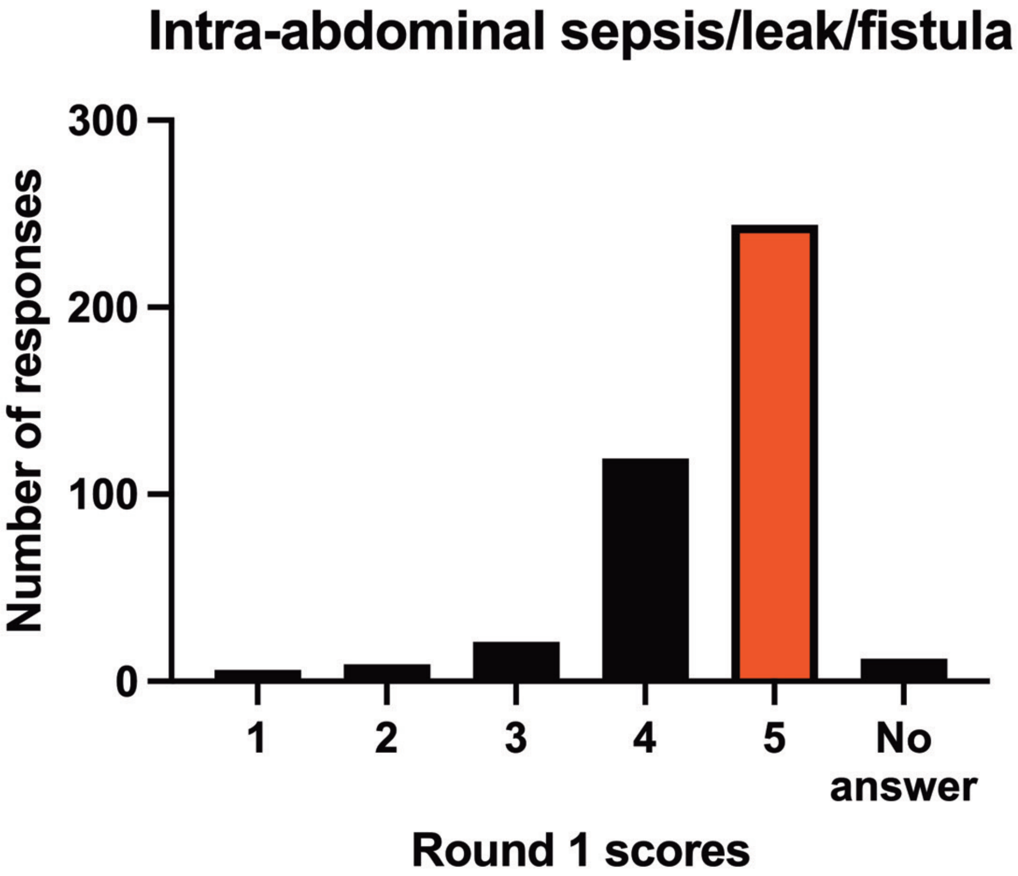


14) Your new answer: "Post-operative intra-abdominal sepsis / anastomotic leak / abscess / fistula" should be incorporated into the Textbook Outcome for emergency TRAUMA laparotomy

Strongly disagree | Neutral | Strongly agree

*(Place a mark on the scale above)*

RESULTS FROM ROUND 1 "Organ failure (or individual organ failures)" should be incorporated into the Textbook Outcome for emergency NON-TRAUMA laparotomy

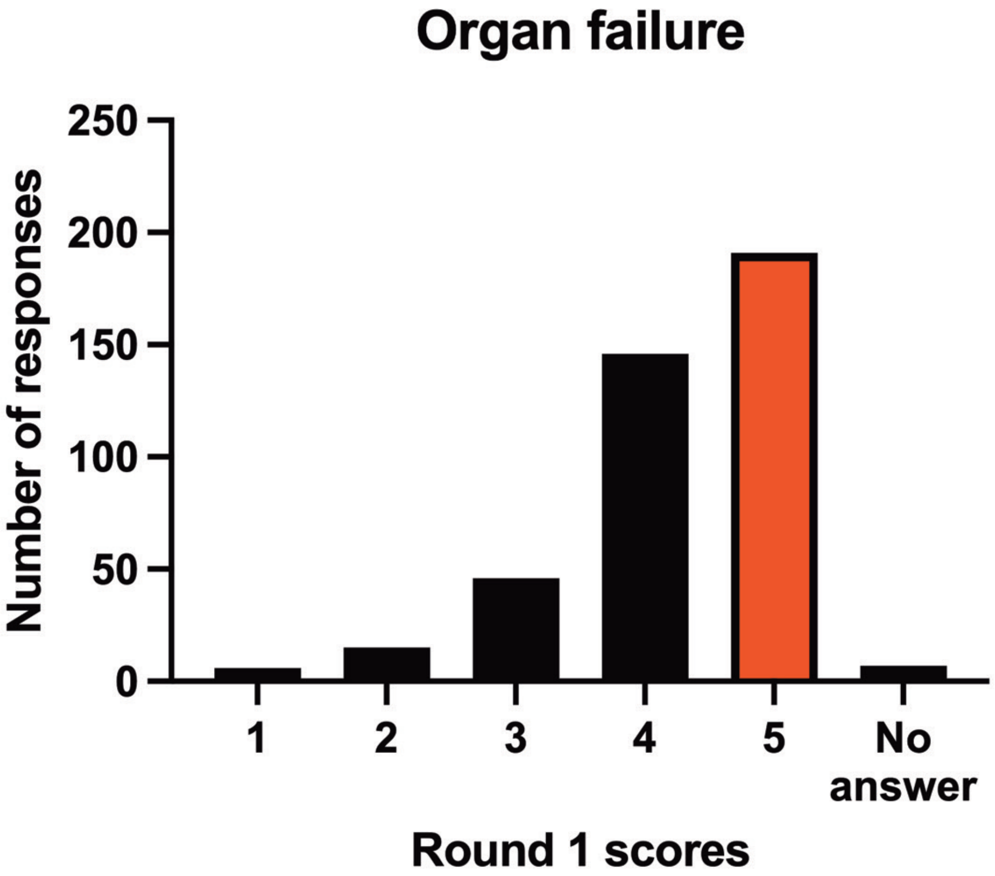


15) Your new answer: "Organ failure (or individual organ failures)" should be incorporated into the Textbook Outcome for emergency NON-TRAUMA laparotomy

Strongly disagree Neutral Strongly agree

_(Place a mark on the scale above)_

RESULTS FROM ROUND 1 "Organ failure (or individual organ failures)" should be incorporated into the Textbook Outcome for emergency TRAUMA laparotomy

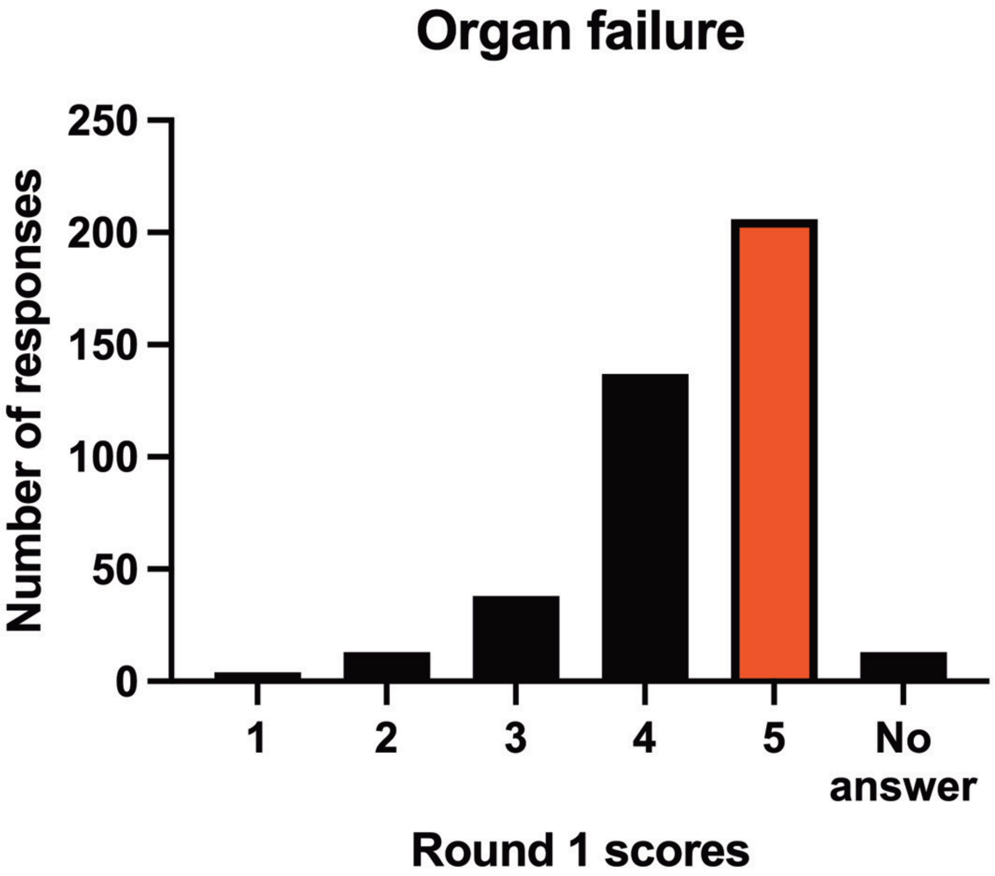


16) Your new answer: "Organ failure (or individual organ failures)" should be incorporated into the Textbook Outcome for emergency TRAUMA laparotomy

Strongly disagree | Neutral | Strongly agree

*(Place a mark on the scale above)*

RESULTS FROM ROUND 1 "Venous thromboembolism (DVT or PE)" should be incorporated into the Textbook Outcome for emergency NON-TRAUMA laparotomy

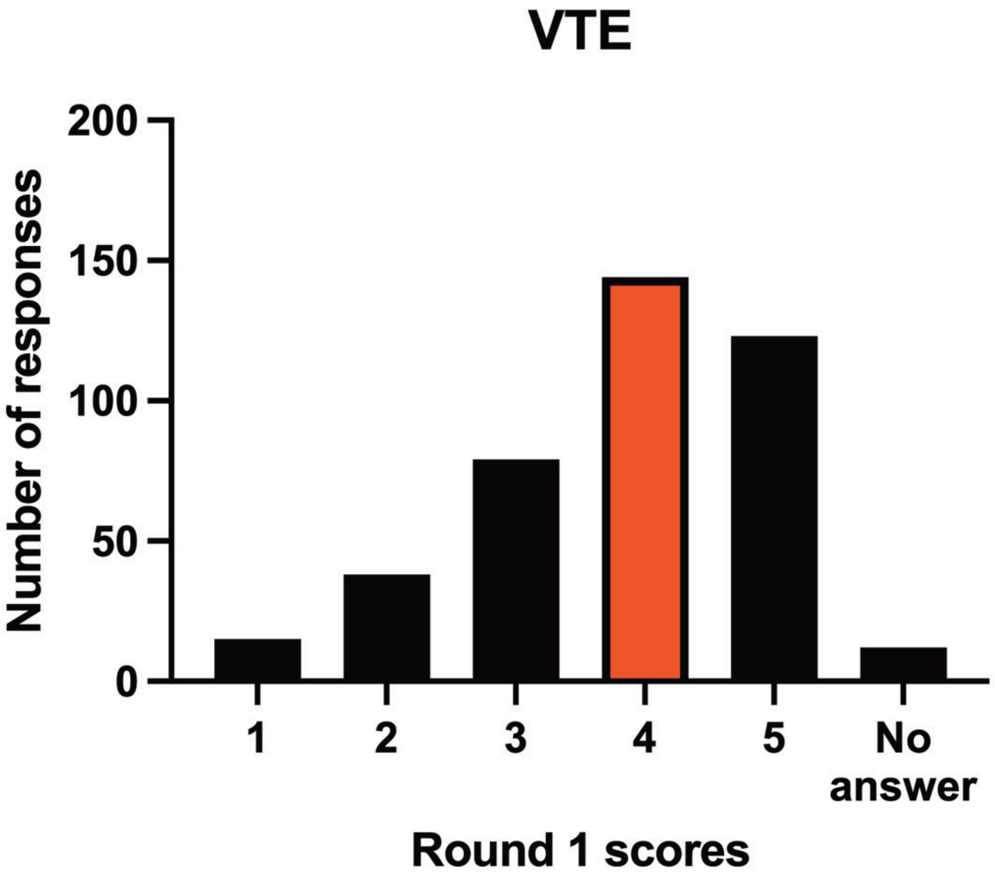


17) Your new answer: "Venous thromboembolism (DVT or PE)" should be incorporated into the Textbook Outcome for emergency NON-TRAUMA laparotomy

Strongly disagree | Neutral | Strongly agree

*(Place a mark on the scale above)*

RESULTS FROM ROUND 1 "Venous thromboembolism (DVT or PE)" should be incorporated into the Textbook Outcome for emergency TRAUMA laparotomy

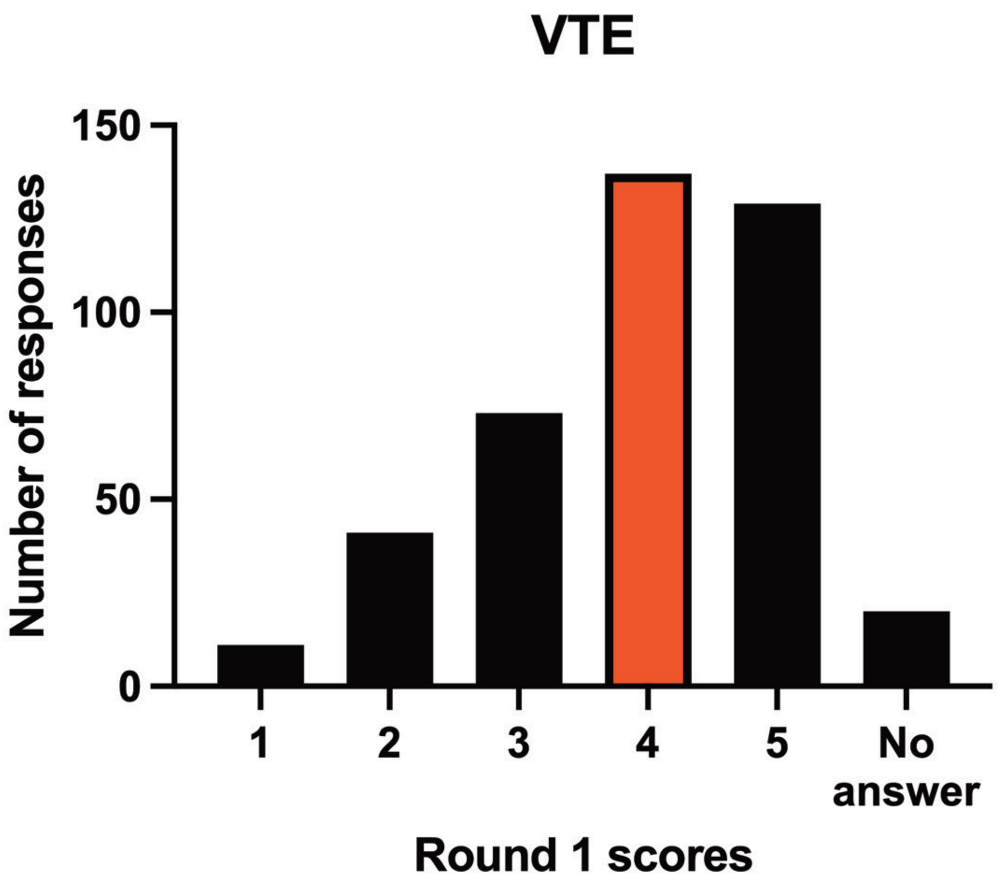


18) Your new answer: "Venous thromboembolism (DVT or PE)" should be incorporated into the Textbook Outcome for emergency TRAUMA laparotomy

Strongly disagree Neutral Strongly agree

_(Place a mark on the scale above)_

RESULTS FROM ROUND 1 "Unplanned re-operation" should be incorporated into the Textbook Outcome for emergency NON-TRAUMA laparotomy

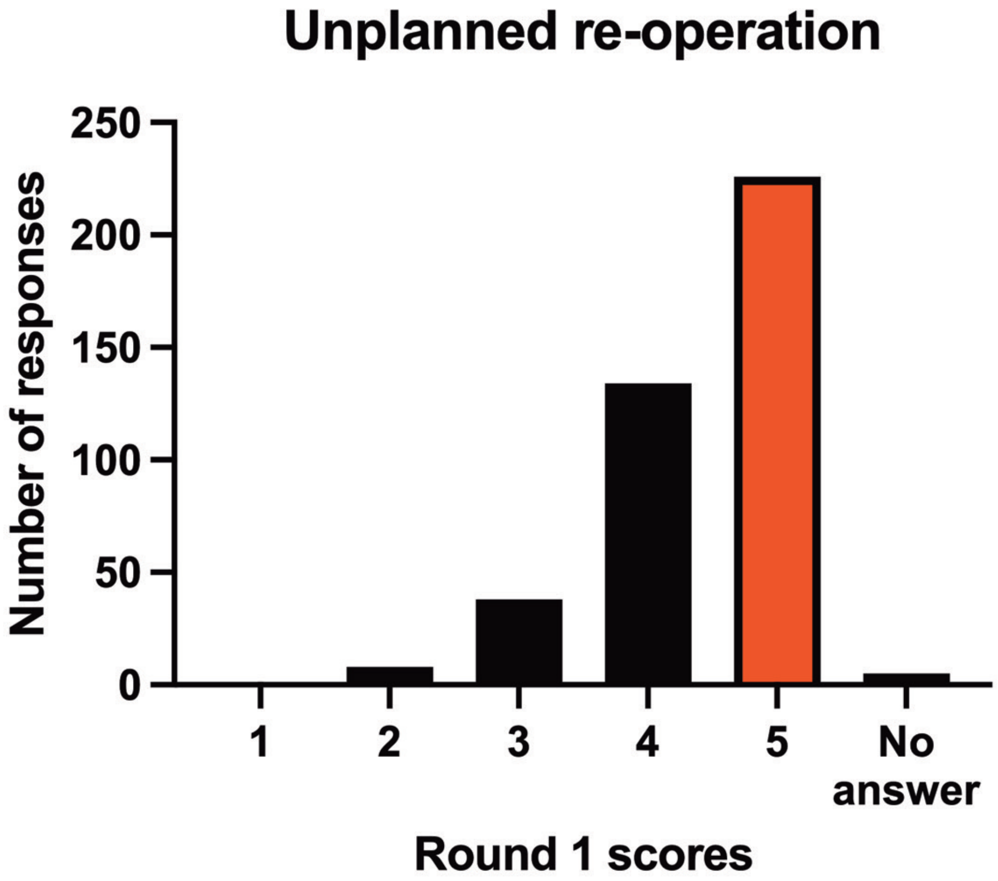


19) Your new answer: "Unplanned re-operation" should be incorporated into the Textbook Outcome for emergency NON-TRAUMA laparotomy

Strongly disagree    Neutral    Strongly agree

*(Place a mark on the scale above)*

RESULTS FROM ROUND 1 "Unplanned re-operation" should be incorporated into the Textbook Outcome for emergency TRAUMA laparotomy

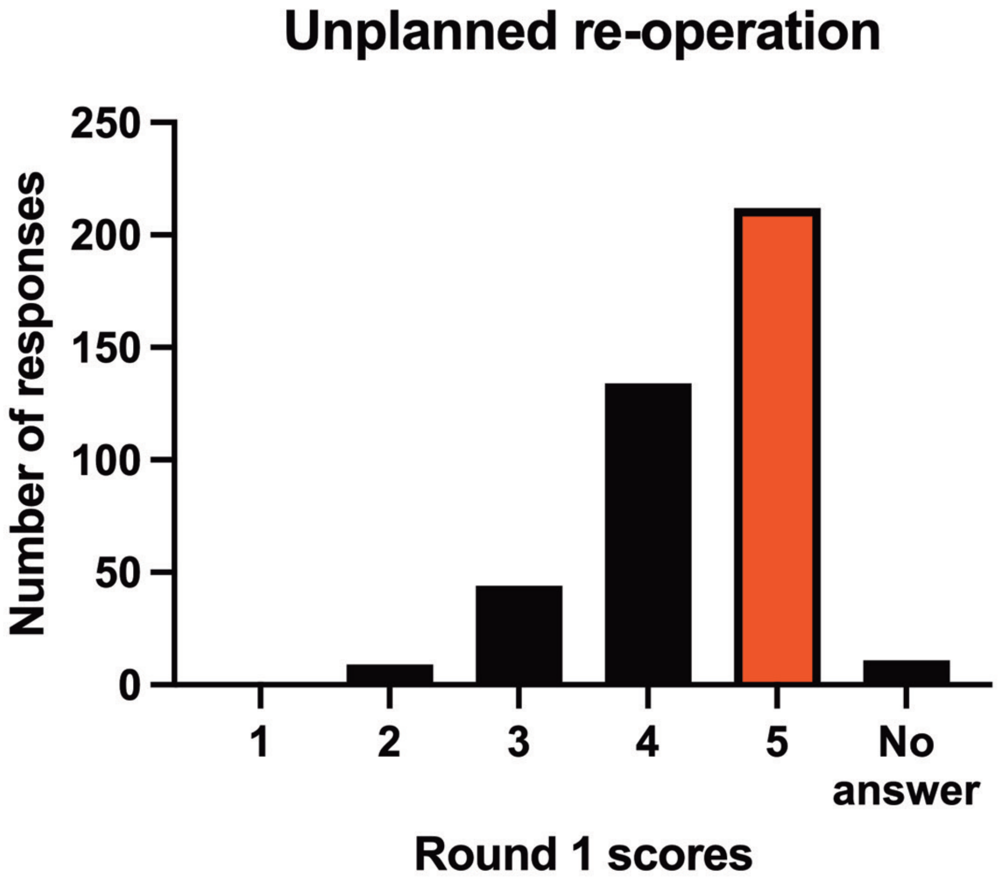


20) Your new answer: "Unplanned re-operation" should be incorporated into the Textbook Outcome for emergency TRAUMA laparotomy

Strongly disagree Neutral Strongly agree

*(Place a mark on the scale above)*

RESULTS FROM ROUND 1 "Post-operative hernia" should be incorporated into the Textbook Outcome for emergency NON-TRAUMA laparotomy

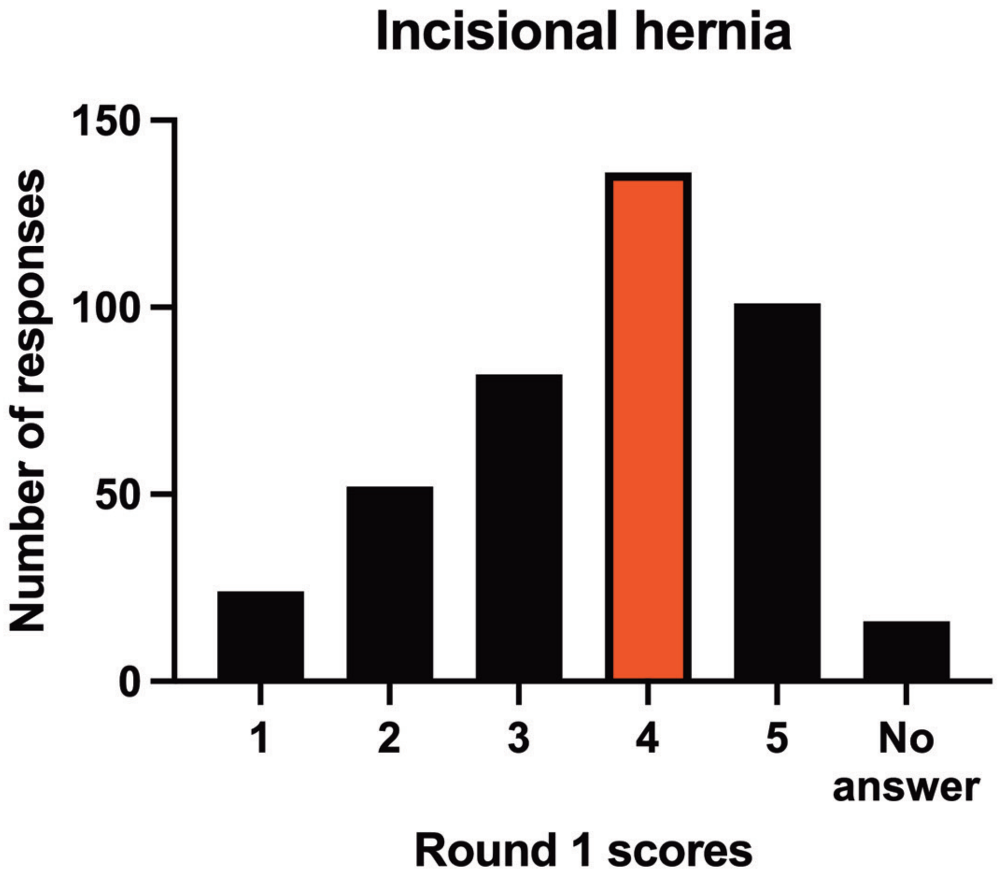


21) Your new answer: "Post-operative hernia" should be incorporated into the Textbook Outcome for emergency NON-TRAUMA laparotomy

Strongly disagree | Neutral | Strongly agree

_(Place a mark on the scale above)_

RESULTS FROM ROUND 1 "Post-operative hernia" should be incorporated into the Textbook Outcome for emergency TRAUMA laparotomy

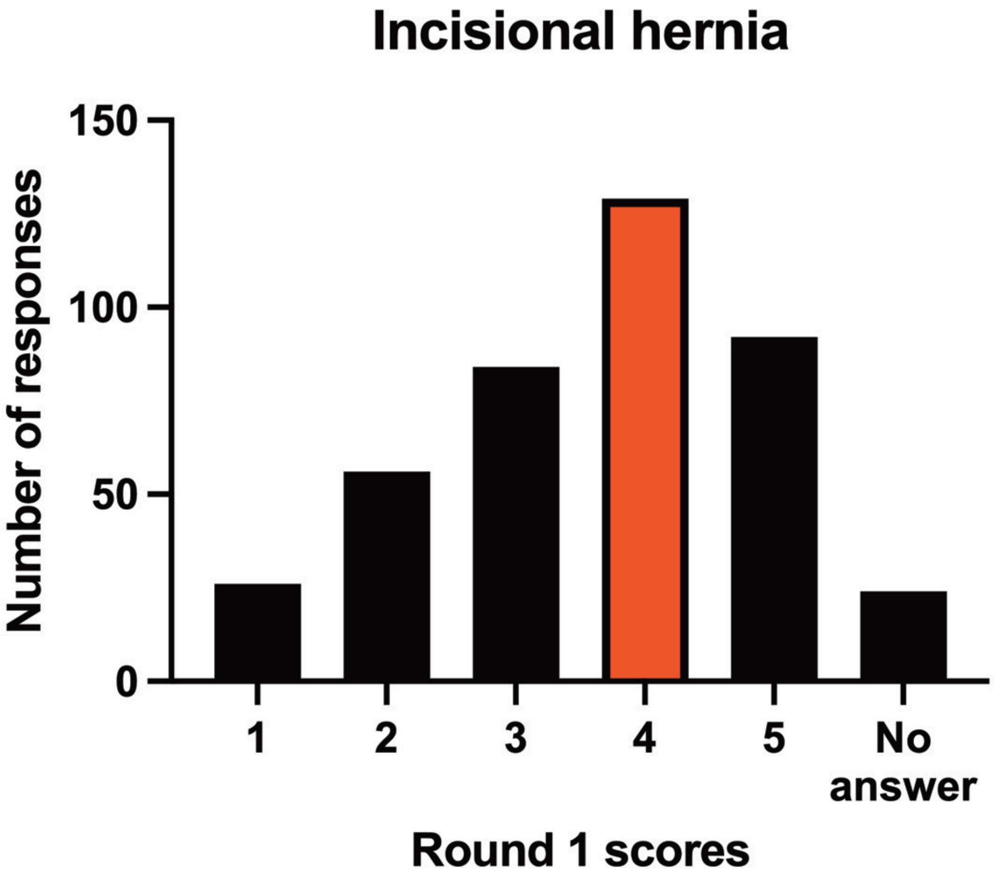


22) Your new answer: "Post-operative hernia" should be incorporated into the Textbook Outcome for emergency TRAUMA laparotomy

Strongly disagree | Neutral | Strongly agree

*(Place a mark on the scale above)*

RESULTS FROM ROUND 1 "Length of stay in hospital" should be incorporated into the Textbook Outcome for emergency NON-TRAUMA laparotomy

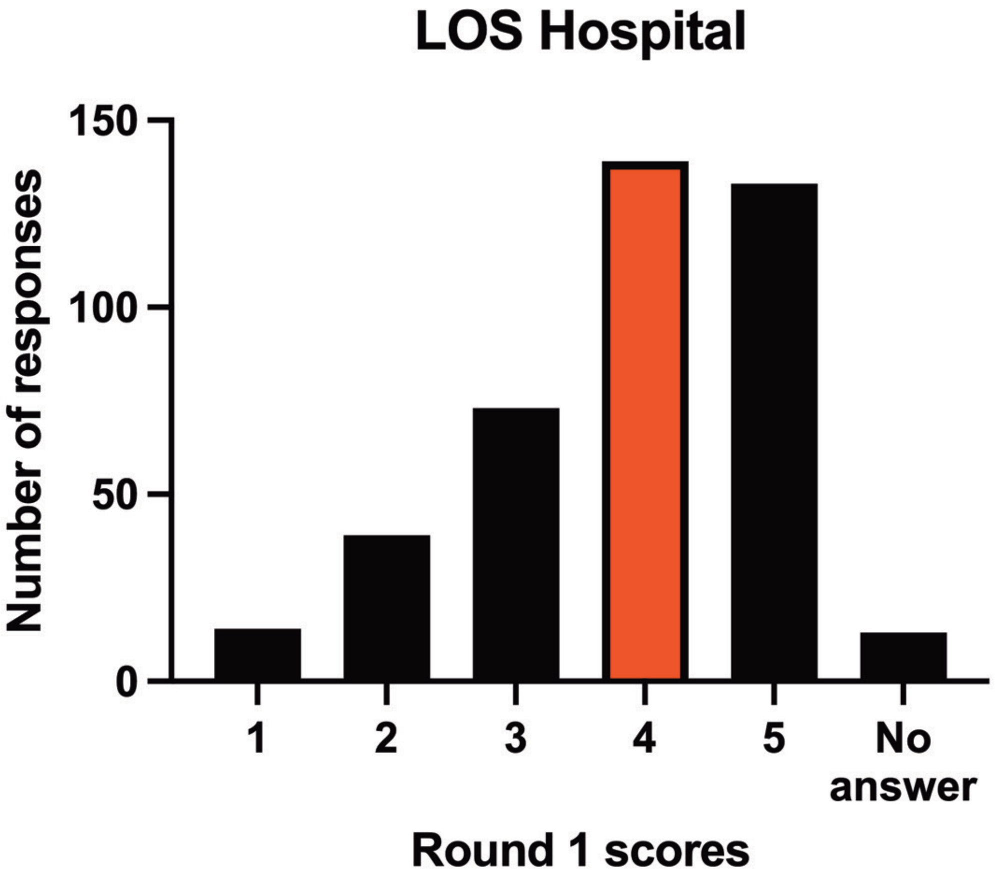


23) Your new answer: "Length of stay in hospital" should be incorporated into the Textbook Outcome for emergency NON-TRAUMA laparotomy

Strongly disagree Neutral Strongly agree

*(Place a mark on the scale above)*

RESULTS FROM ROUND 1 "Length of stay in hospital" should be incorporated into the Textbook Outcome for emergency TRAUMA laparotomy

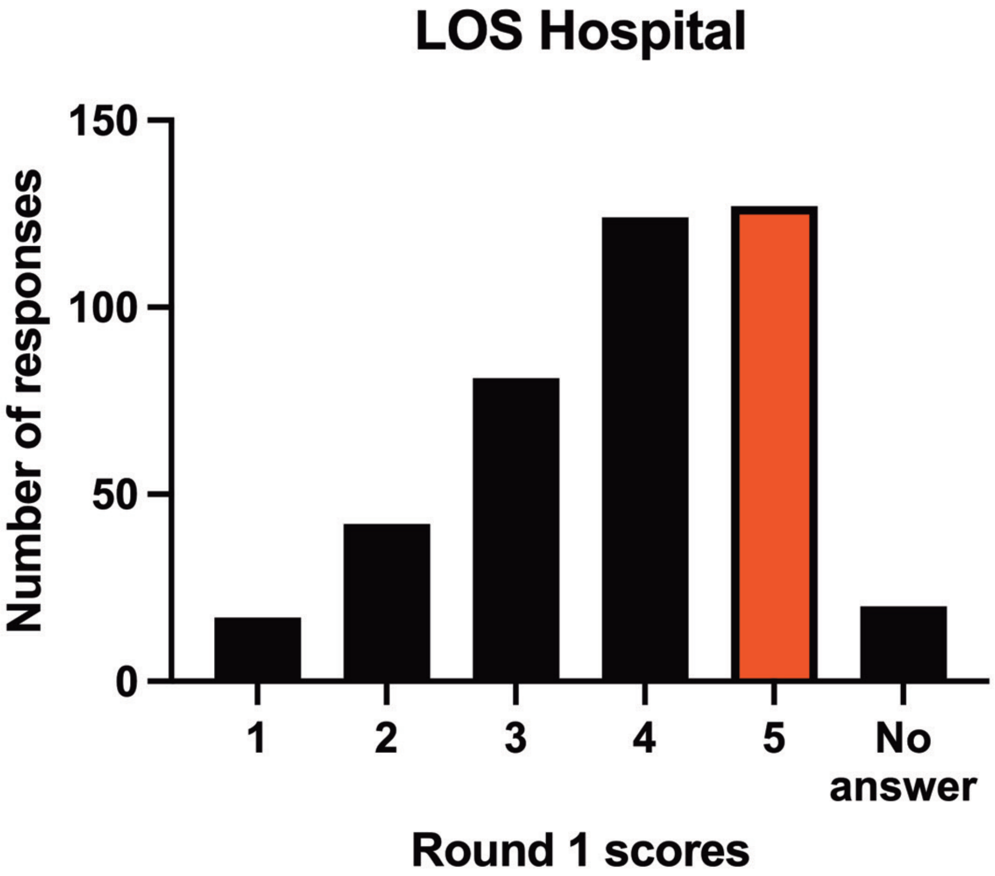


24) Your new answer: "Length of stay in hospital" should be incorporated into the Textbook Outcome for emergency TRAUMA laparotomy

Strongly disagree Neutral Strongly agree

_(Place a mark on the scale above)_

RESULTS FROM ROUND 1 "Length of stay in the Intensive Care Unit" should be incorporated into the Textbook Outcome for emergency NON-TRAUMA laparotomy

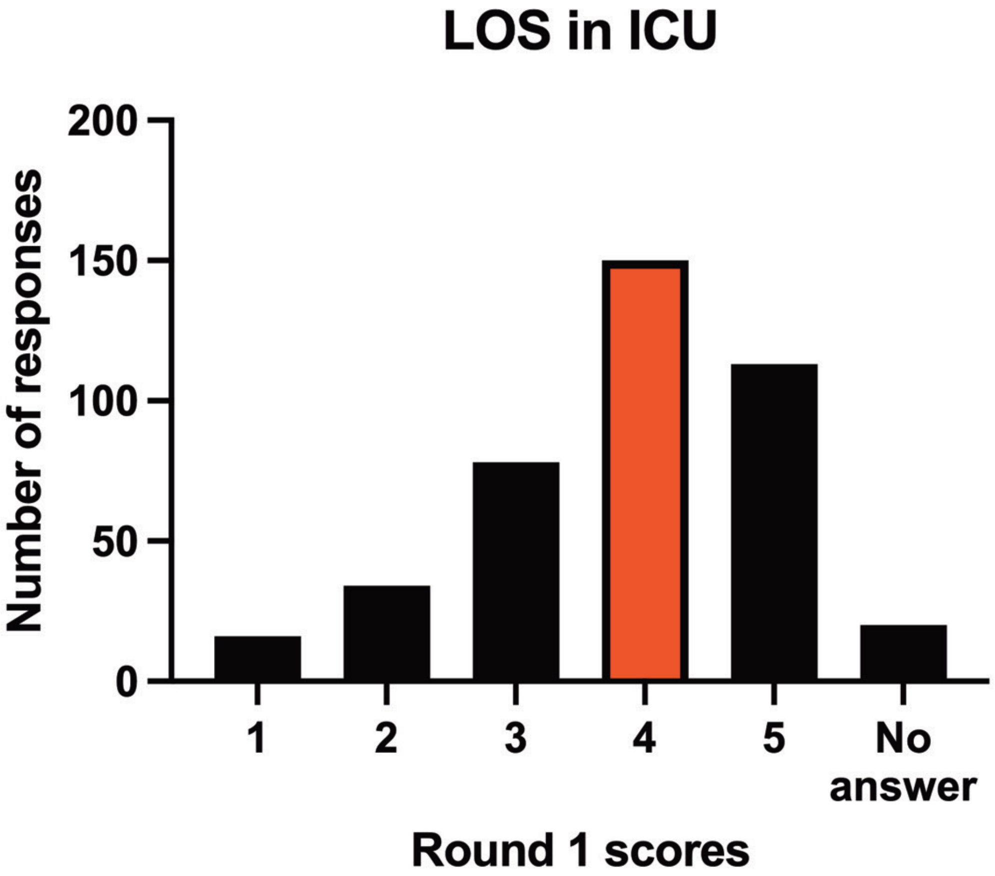


25) Your new answer: "Length of stay in the Intensive Care Unit" should be incorporated into the Textbook Outcome for emergency NON-TRAUMA laparotomy

Strongly disagree Neutral Strongly agree

*(Place a mark on the scale above)*

RESULTS FROM ROUND 1 "Length of stay in the Intensive Care Unit" should be incorporated into the Textbook Outcome for emergency TRAUMA laparotomy

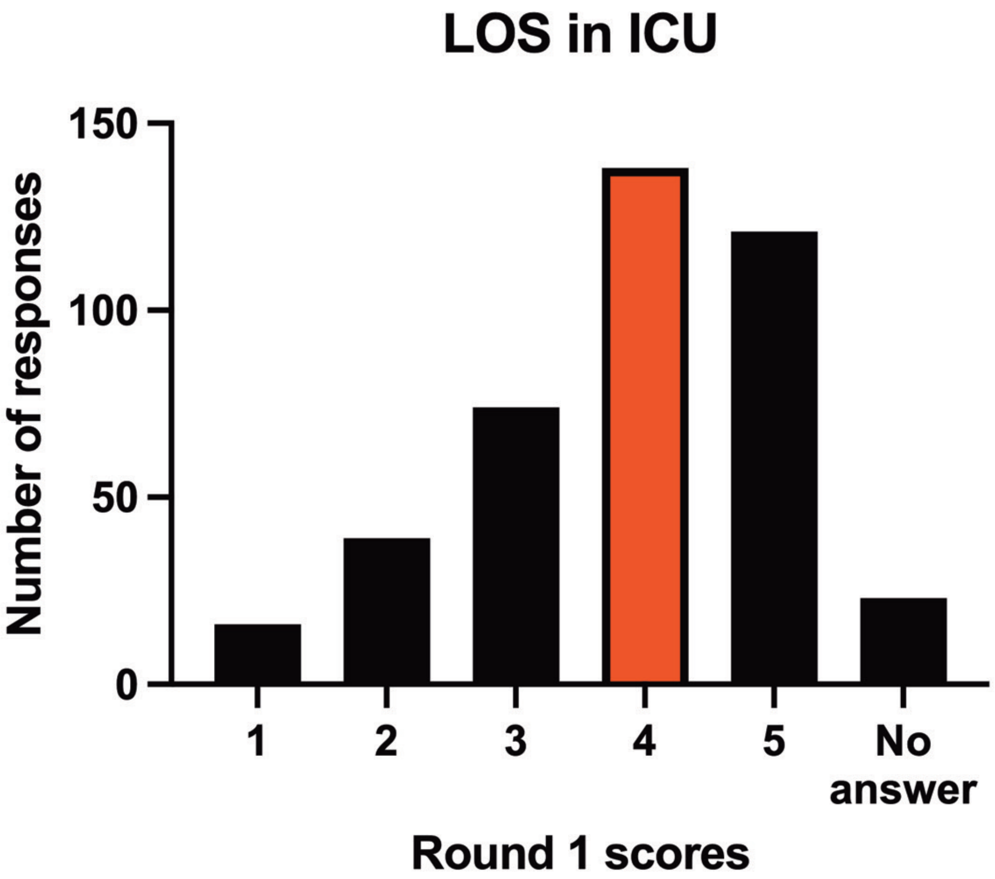


26) Your new answer: "Length of stay in the Intensive Care Unit" should be incorporated into the Textbook Outcome for emergency TRAUMA laparotomy

Strongly disagree | Neutral | Strongly agree

*(Place a mark on the scale above)*

RESULTS FROM ROUND 1 A form of functional or quality of life outcome should be incorporated into the Textbook Outcome for emergency NON-TRAUMA laparotomy

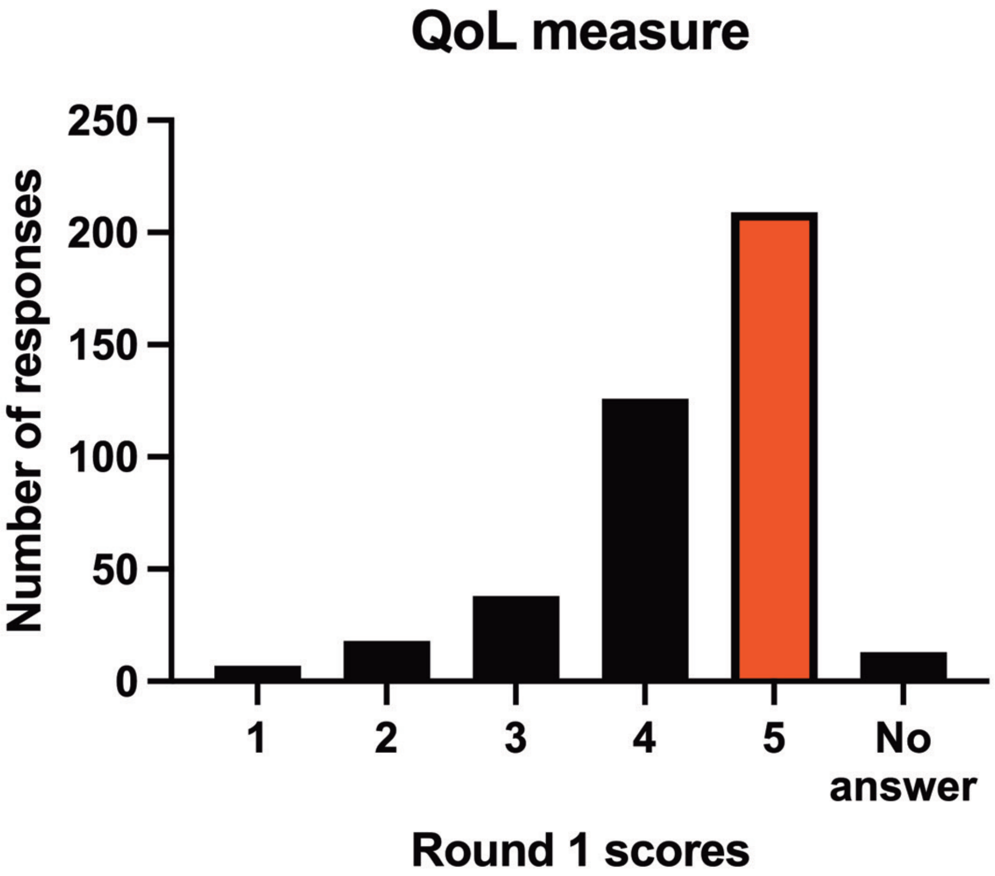


27) Your new answer: A form of functional or quality of life outcome should be incorporated into the Textbook Outcome for emergency NON-TRAUMA laparotomy

Strongly disagree Neutral Strongly agree

_(Place a mark on the scale above)_

RESULTS FROM ROUND 1 A form of functional or quality of life outcome should be incorporated into the Textbook Outcome for emergency TRAUMA laparotomy

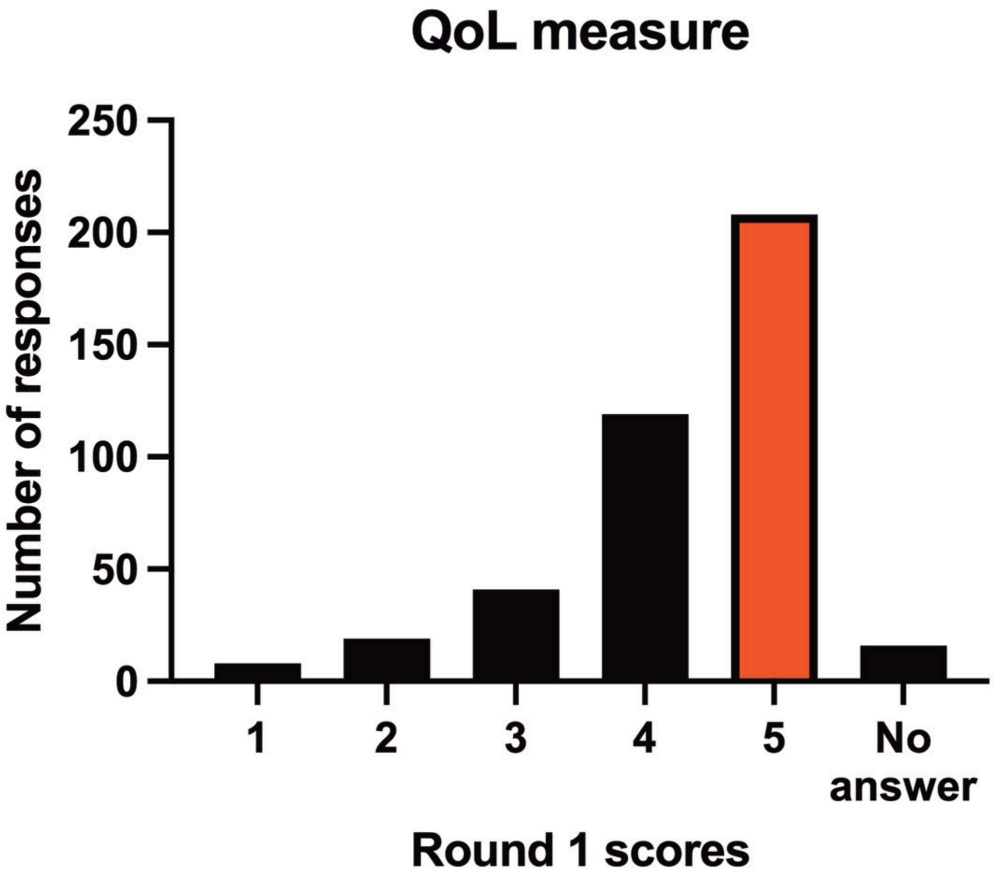


28) Your new answer: A form of functional or quality of life outcome should be incorporated into the Textbook Outcome for emergency TRAUMA laparotomy

Strongly disagree Neutral Strongly agree

*(Place a mark on the scale above)*

RESULTS FROM ROUND 1 Requirement for ongoing rehabilitation should be incorporated into the Textbook Outcome for emergency NON-TRAUMA laparotomy

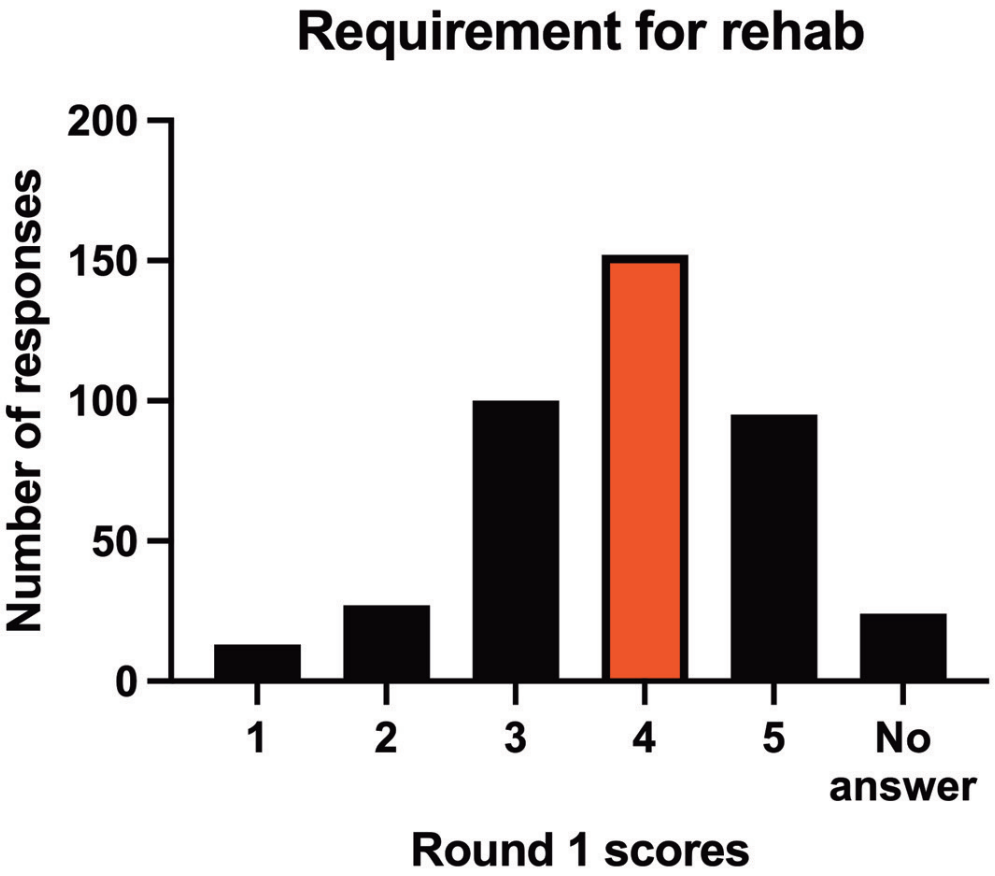


29) Your new answer: Requirement for ongoing rehabilitation should be incorporated into the Textbook Outcome for emergency NON-TRAUMA laparotomy

Strongly disagree | Neutral | Strongly agree

*(Place a mark on the scale above)*

RESULTS FROM ROUND 1 Requirement for ongoing rehabilitation should be incorporated into the Textbook Outcome for emergency TRAUMA laparotomy

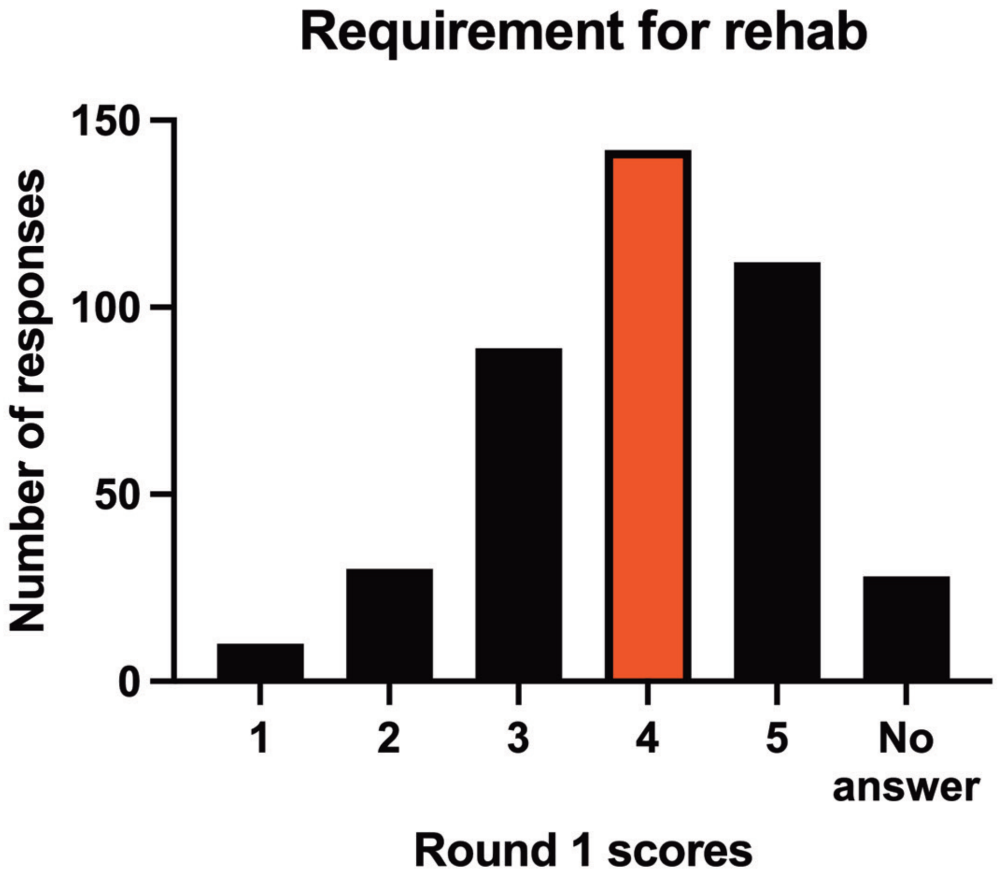


30) Your new answer: Requirement for ongoing rehabilitation should be incorporated into the Textbook Outcome for emergency TRAUMA laparotomy

Strongly disagree Neutral Strongly agree

*(Place a mark on the scale above)*

RESULTS FROM ROUND 1 "Return to work" should be incorporated into the Textbook Outcome for emergency NON-TRAUMA laparotomy

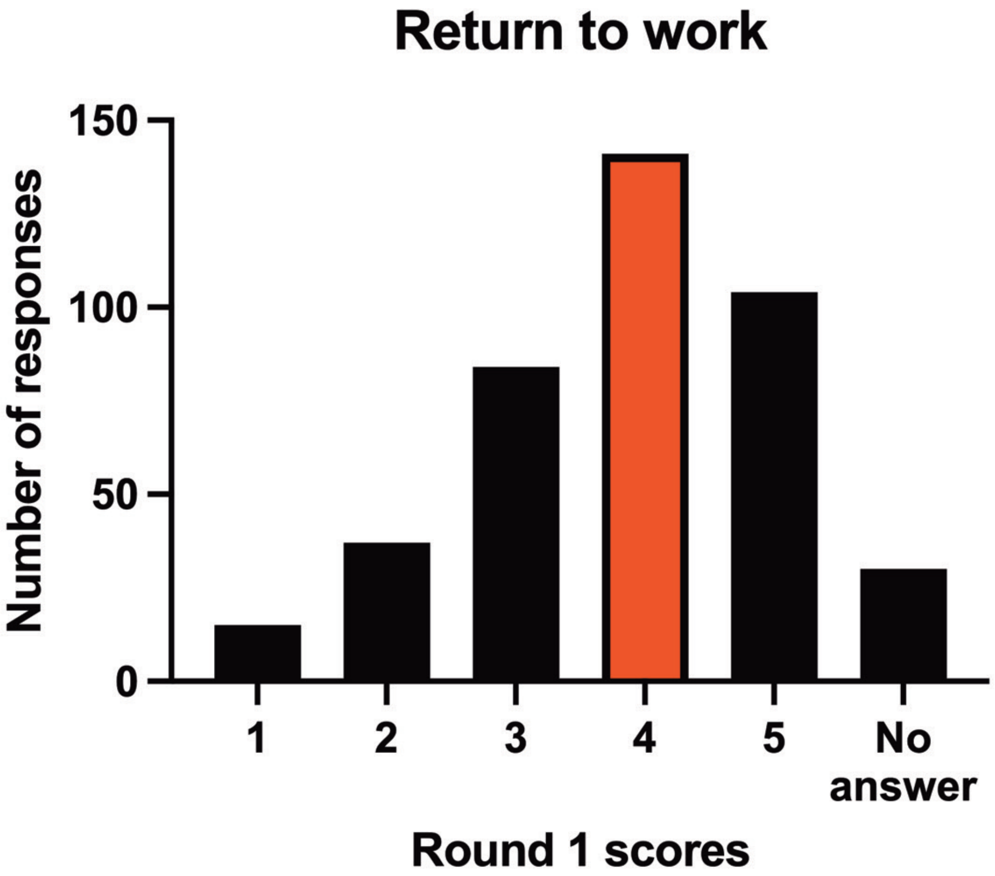


31) Your new answer: "Return to work" should be incorporated into the Textbook Outcome for emergency NON-TRAUMA laparotomy

Strongly disagree | Neutral | Strongly agree

*(Place a mark on the scale above)*

RESULTS FROM ROUND 1 "Return to work" should be incorporated into the Textbook Outcome for emergency TRAUMA laparotomy

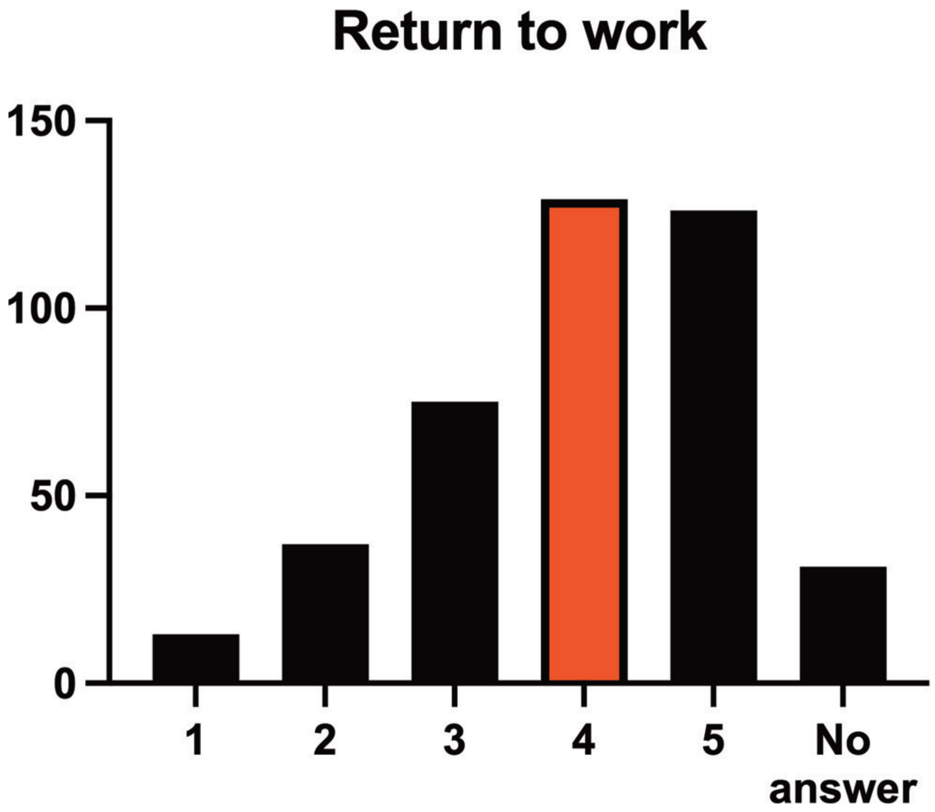


32) Your new answer: "Return to work" should be incorporated into the Textbook Outcome for emergency TRAUMA laparotomy

Strongly disagree Neutral Strongly agree

_(Place a mark on the scale above)_

RESULTS FROM ROUND 1 Follow up period should be:

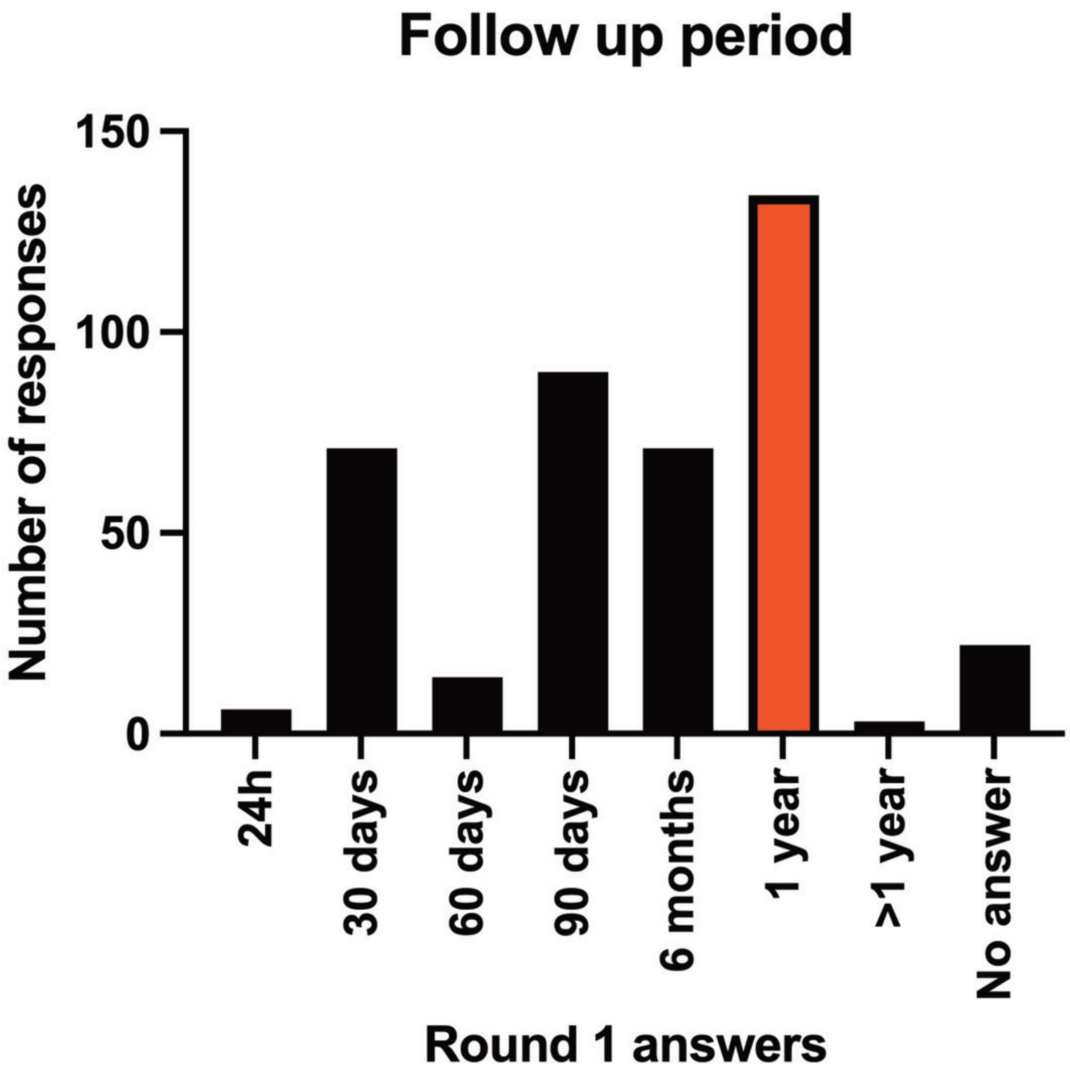


33) Your new answer: Follow up period should be:

◯ 24 hours ◯ 30 days
◯ 60 days ◯ 90 days
◯ 6 months ◯ 1 year

RESULTS FROM ROUND 1 How many individual outcomes do you believe can realistically be combined in a composite Textbook Outcome to be practical and useful?

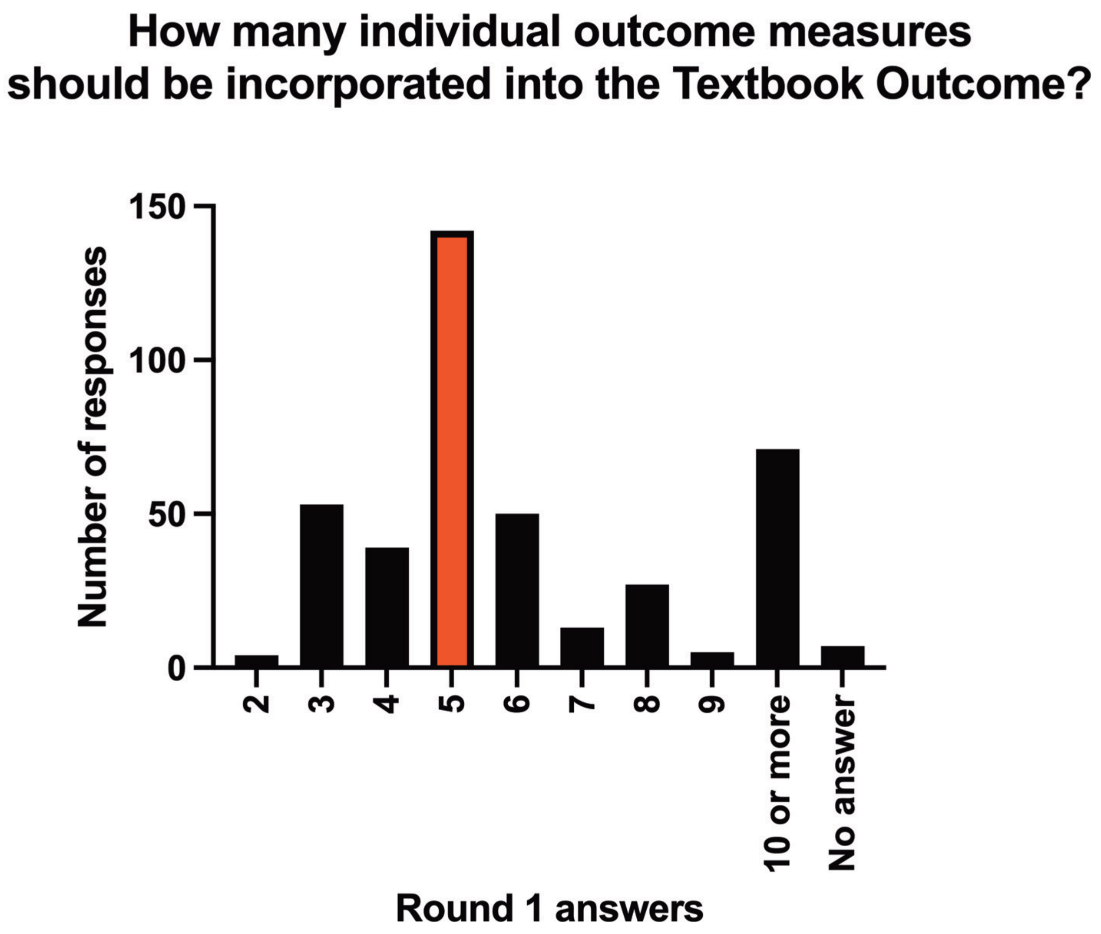


34) Your new answer: How many individual outcomes do you believe can realistically be combined in a composite Textbook Outcome to be practical and useful?

◯ 2 ◯ 3 ◯ 4 ◯ 5
◯ 6 ◯ 7 ◯ 8 ◯ 9
◯ 10 or more

35) If only the top (red bar chart) answers from Round 1 were used to form a Textbook Outcome for NON-TRAUMA emergency laparotomy, it would be something like this: “Discharged without any post-operative complication, unplanned re-operation or organ failure, with good quality of life at 1 year” Would you agree that this may be the best Textbook Outcome for emergency NON-TRAUMA laparotomy?

◯ Yes
◯ No
◯ I will comment below

36) Further comments for emergency NON-TRAUMA laparotomy

__________________________________

| | | |
|---|---|---|
| 37) | If only the top (red bar chart) answers from Round 1 were used to form a Textbook Outcome for TRAUMA emergency laparotomy, it would be something like this: “Discharged within 30 days without any post-operative complication, unplanned re-operation, organ failure or unplanned transfusion, with good quality of life at 1 year” Would you agree that this may be the best Textbook Outcome for emergency TRAUMA laparotomy? | ◯ Yes<br>◯ No<br>◯ I will comment below |
| 38) | Further comments for emergency TRAUMA laparotomy | __________________________________ |
| 39) | I would be happy to participate in a third (final) round of this Delphi process and be listed as a co-author | ◯ Yes<br>◯ No, please only list me in the Acknowledgments section<br>◯ I wish to withdraw from this process completely |

**Supplementary Results 3. Delphi Round 3**



# ROUND 3 (final round) of Delphi - Textbook Outcomes following emergency laparotomy

Please complete the survey below.

Thank you!

---

1) Round 3 (final round) of the Delphi process:

Textbook Outcomes for Trauma and Emergency General Surgery

Thank you for your input so far! Please complete Round 3 (final round) in order to be eligible for authorship in the final publication. Reminder:
1. What are Textbook Outcomes? "Textbook Outcomes" are composite measures that incorporate multiple patient outcomes representing the "ideal" or best possible outcome. Rather than an individual outcome such as "survival" after cancer resection, an example of a Textbook Outcome may be "returned home alive and without any surgical complications, with a R0 resection and appropriately radical lymphadenectomy on histology".

2. What is the purpose of this Delphi Exercise? This Delphi exercise seeks to define which Textbook Outcomes might be the best to use for patients following emergency laparotomy, based on your expert opinion[1].

3. What will be done with my responses? Your anonymous, voluntary responses to this THIRD round of questions will be used to achieve overall consensus amongst experts in their field.

4. References 1. Naumann DN, Bhangu A, Brooks A, Martin M, Cotton BA, Khan M, Midwinter MJ, Pearce L, Bowley DM, Holcomb JB, Griffiths EA. A call for patient-centred textbook outcomes for emergency surgery and trauma. Br J Surg. 2022 (https://academic.oup.com/bjs/advance-article/doi/10.1093/bjs/znac271/6670913?searchresult=1)

◯ I understand all of this and wish to proceed with the survey

---

2) Please confirm your full name as you would like it to appear in the authorship (include middle initial(s) if appropriate) ______________________________

---

3) Please confirm your affiliation as you would like it to appear in the published article (please include full address including country) ______________________________

---

4) Please confirm your email address here, so that we can confirm your participation and prepare for the third (final) round of the Delphi ______________________________

Please read the results (1-3) below and then respond to the questions:

5) RESULTS (1 of 3)
These are how many individual outcomes participants thought would be ideal: Overall, the most common answer was "5"

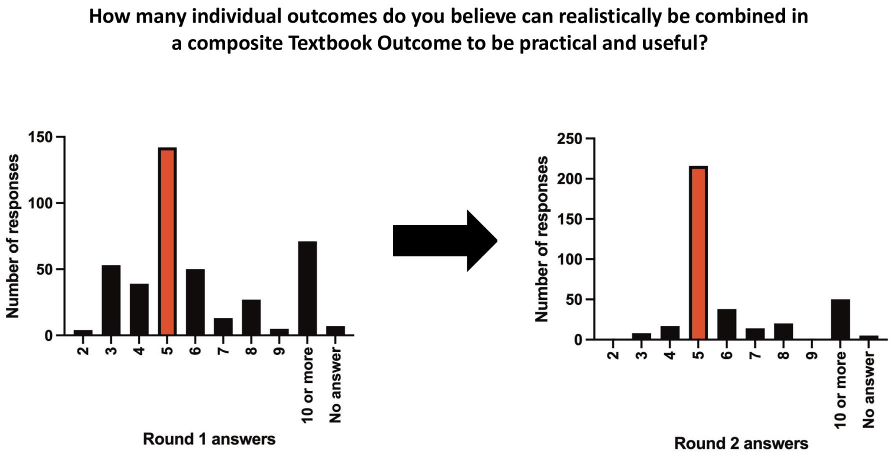


5) RESULTS (2 of 3): These are the answers for how long follow up should go up to. Overall, 1 year was the most popular answer

**Follow up period should be:**

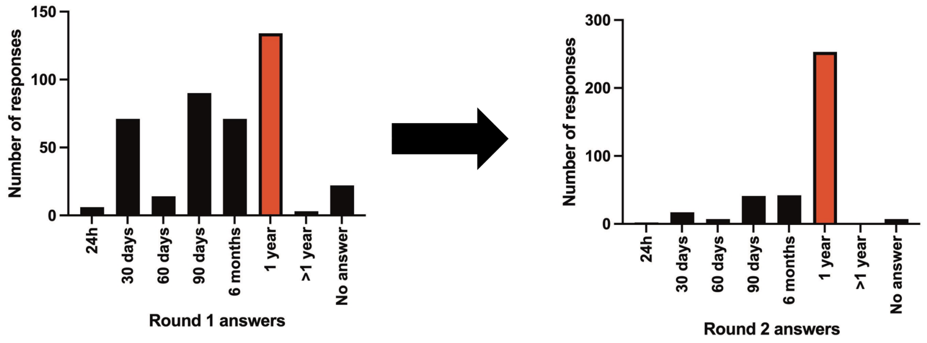


5) RESULTS (3 of 3): These are the individual outcome measures in order of popularity according to number of participants grading as a "5" (maximum) on the Likert scale.    Text highlighted in yellow  indicates more than half the respondents graded a "5"

**Non-trauma laparotomy**

| Outcome | Responses as 5 |
|---|---|
| Post-op complications | 334 |
| Mortality/survival | 325 |
| Intra-abdo sepsis/fistula/leak/abscess | 320 |
| Unplanned re-operation | 302 |
| Organ failure | 280 |
| Quality of life | 276 |
| LOS in hospital | 134 |
| LOS in ICU | 117 |
| VTE | 109 |
| Fascial closure | 108 |
| Post-op hernia | 96 |
| Return to work | 91 |
| 'Blood products used' | 68 |
| Ongoing rehabilitation | 68 |
| Time to normalise lactate | 42 |

**Trauma laparotomy**

| Outcome | Responses as 5 |
|---|---|
| Post-op complications | 329 |
| Mortality/survival | 325 |
| Intra-abdo sepsis/fistula/leak/abscess | 308 |
| Unplanned re-operation | 300 |
| Organ failure | 286 |
| Quality of life | 276 |
| Blood products used | 224 |
| LOS in hospital | 155 |
| VTE | 121 |
| LOS in ICU | 117 |
| Return to work | 115 |
| Fascial closure | 111 |
| Ongoing rehabilitation | 86 |
| Post-op hernia | 78 |
| Time to normalise lactate | 54 |

5) Our patient focus group suggested that having a combination of a short term and a long term Textbook Outcome would be superior than just one overall Textbook Outcome. Do you agree that this is a good idea?

◯ Yes ◯ No ◯ I will comment below

6) Further comments:

__________________________________________

7) Our patient focus group suggested that they would prefer the Quality of Life measure to be restoration of baseline quality of life (rather than lengthy or detailed questionaires). Do you agree that this is a good idea?

◯ Yes ◯ No ◯ I will comment below

8) Further comments:

__________________________________________

9) For NON-TRAUMA Laparotomy: Based on these answers, to what extent do you agree that the "Early" and "Longer Term" Textbook Outcomes should be:

Early Textbook Outcome (E-TO): Discharged from hospital without serious post-operative complications (i.e. Clavien-Dindo* ≥ grade 3; including intra-abdominal sepsis, organ failure, unplanned re-operation or death).

Longer Term Textbook Outcome (LT-TO): Achieved the E-TO, and also restoration of baseline quality of life at 1 year.

Reference: *Dindo D, Demartines N, Clavien PA. Classification of surgical complications: a new proposal with evaluation in a cohort of 6336 patients and results of a survey. Ann Surg. 2004 Aug;240(2):205-13. https://www.ncbi.nlm.nih.gov/pmc/articles/PMC1360123/

◯ Yes I agree ◯ I would like to suggest something different (see comment below)

10) Further comments or suggested edits:

__________________________________________

11) For TRAUMA Laparotomy: Based on these answers, to what extent do you agree that the "Early" and "Longer Term" Textbook Outcomes should be:

Early Textbook Outcome (E-TO): "Discharged from hospital without unexpected transfusion after haemostasis, and no serious post-operative complications (Adapted Clavien-Dindo for Trauma* ≥ grade 3; including intra-abdominal sepsis, organ failure, unplanned re-operation or death)."

Longer Term Textbook Outcome (LT-TO): "Achieved the E-TO, and also restoration of baseline quality of life at 1 year." Reference: *Naumann DN, Vincent LE, Pearson N, Beaven A, Smith IM, Smith K, Toman E, Dorrance HR, Porter K, Wade CE, Cotton BA, Holcomb JB, Midwinter MJ. An adapted Clavien-Dindo scoring system in trauma as a clinically meaningful nonmortality endpoint. J Trauma Acute Care Surg. 2017 Aug;83(2):241-248. https://journals.lww.com/jtrauma/Abstract/2017/08000/An_adapted_Clavien_Dindo_scoring_system_in_trauma.6.aspx

◯ Yes I agree ◯ I would like to suggest something different (see comment below)

12) Further comments or suggested edits:

______________________________________

**Supplementary Table 1. Full list of countries of participants**

| Country | Number of participants |
|---|---|
| Albania | 1 |
| Argentinian | 1 |
| Australia | 15 |
| Austria | 1 |
| Belarus | 2 |
| Belgium | 3 |
| Brazil | 2 |
| Bulgaria | 2 |
| Canada | 7 |
| Chile | 1 |
| China | 6 |
| Colombia | 1 |
| Croatia | 1 |
| Denmark | 11 |
| Finland | 5 |
| France | 6 |
| Georgia | 1 |
| Germany | 4 |
| Ghana | 1 |
| Greece | 14 |
| Hungary | 1 |
| India | 10 |
| Iran | 2 |
| Ireland | 2 |
| Israel | 3 |
| Italy | 55 |
| Japan | 3 |
| Latvia | 1 |
| Malaysia | 1 |
| Mexico | 2 |
| Nepal | 1 |
| Netherlands | 7 |
| New Zealand | 1 |
| Nigeria | 3 |
| Poland | 1 |
| Portugal | 2 |
| Romania | 3 |
| Russian Federation | 1 |
| Saudi Arabia | 2 |
| Serbia | 1 |

| | |
|---|---|
| Singapore | 2 |
| South Africa | 3 |
| South Korea | 1 |
| Spain | 3 |
| Sweden | 3 |
| Switzerland | 3 |
| Tunisia | 2 |
| Turkey | 6 |
| Ukraine | 1 |
| United Arab Emirates | 2 |
| United Kingdom | 68 |
| United States | 55 |
| Vietnam | 1 |
